# A Hf(IV)‐Coordinated NIR‐II Fluorescence “Turn‐On” Nanoprobe for Precise Imaging‐Guided Surgery in Breast Cancer

**DOI:** 10.1002/advs.202413385

**Published:** 2025-02-14

**Authors:** Yueyang He, Jinyan Lin, Jingwen Bai, Xiao Shen, Kangliang Lou, Yuanyuan Zhu, Zishan Qiao, Weiling Chen, Yang Li, Xiaolong Liu, Guojun Zhang

**Affiliations:** ^1^ Fujian Key Laboratory of Precision Diagnosis and Treatment in Breast Cancer Xiang'an Hospital of Xiamen University School of Medicine Xiamen University Xiamen 361100 P. R. China; ^2^ The United Innovation of Mengchao Hepatobiliary Technology Key Laboratory of Fujian Province Mengchao Hepatobiliary Hospital of Fujian Medical University Fuzhou 350025 P. R. China; ^3^ The Breast Center Yunnan Cancer Hospital & The Third Affiliated Hospital of Kunming Medical University & Yunnan Cancer Center Kunming 650118 P. R. China; ^4^ State Key Laboratory of Structural Chemistry & Xiamen Institute of Rare Earth Materials, Fujian Institute of Research on the Structure of Matter Chinese Academy of Sciences Fuzhou 350002 P. R. China

**Keywords:** breast cancer, imaging‐guided surgery, molecular probes, NIR‐II fluorescence

## Abstract

Breast‐conserving surgery (BCS) has become the standard care for early‐stage breast cancer. The accurate assessment of tumor margins is urgently required for BCS because positive resection margins often lead to local recurrence. To address this clinical dilemma, a Hf(IV)‐coordinated NIR‐II fluorescence “turn‐on” nanoprobe based on the clinically approved NIR‐II fluorescent dye indocyanine green (ICG) for intraoperative tumor visualization is developed. Notably, the fluorescence of ICG can be efficiently quenched by Hf(IV) and subsequently recovered in vivo, showing a remarkable fluorescence “quenching‐recovery‐amplification” capacity. This nanoprobe can effectively accumulate in tumor sites, accurately identifying submillimeter‐sized primary and residual tumors with high sensitivity. In addition, subcutaneous, muscle‐infiltrating, and orthotopic breast cancer models are built to repeatedly prove that this ultrasensitive nanoprobe is feasible for precise imaging‐guided surgery in breast cancer. Overall, this study constructs an activatable fluorescent nanoprobe for real‐time intraoperative tumor margin visualization, holding promise for complete surgical resection and reduction of local recurrence.

## Graphical abstract

Based on the FDA‐approved fluorescent dye ICG, a Hf(IV)‐coordinated NIR‐II fluorescence “turn‐on” nanoprobe is constructed. This ultrasensitive nanoprobe can effectively accumulate in tumor sites and recover the fluorescence of ICG specifically in tumor microenvironment, accurately identifying submillimeter‐sized tumors with high sensitivity for real‐time intraoperative tumor margin visualization and complete surgical resection.

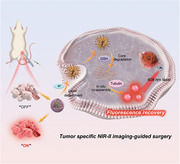

## 1 Introduction

Breast‐conserving surgery (BCS) is typically recommended for early‐stage breast cancer patients.^[^ ^1^ ^]^ Margin assessment, which requires accurately identifying the tumor and healthy tissue, is crucial during BCS as the positive resection margins often lead to local recurrence.^[^ ^2^ ^]^ However, the accuracy of intraoperative tumor margin identification is constrained by its reliance on palpation and visual inspection with inconsistency and a rough estimation. Intraoperative frozen section analysis and imprint cytology help to evaluate tumor margins during surgery.^[^ ^3^ ^]^ However, these methods fail to accurately represent intact tissue and often result in a 5–15% discrepancy with postoperative pathology findings and extend the duration of the surgery.^[^ ^3^, ^4^ ^]^ Additionally, conventional imaging methods like X‐ray radiography, computed tomography, and magnetic resonance imaging possess restricted sensitivity, specificity, or ionizing radiation, making them challenging in surgical navigation.^[^ ^5^ ^]^

Fluorescence imaging, especially in the second near‐infrared (NIR‐II) window (1000–1700 nm), has shown great potential for real‐time intraoperative tumor margin visualization with high sensitivity, high resolution, deep penetration depth, and fast feedback.^[^ ^6^ ^]^ So far, various NIR‐II fluorescent dyes have been investigated for surgical navigation.^[^ ^7^ ^]^ Notably, the clinically approved dye ICG exhibits off‐peak emission spectra in the NIR‐II region with appreciable quantum yield,^[^ ^8^ ^]^ which has been extensively explored.^[^ ^9^ ^]^ However, evidence demonstrated that ICG showed poor tumor enrichment efficiency, making it hard to distinguish the tumor lesions from adjacent normal tissue by NIR‐II imaging.^[^ ^7^, ^10^ ^]^ In addition, during surgery, as remaining tumor lesions get smaller and smaller, intraoperative imaging with higher specificity and sensitivity is required.^[^ ^11^ ^]^ To improve the tumor accumulation efficiency and imaging effect of ICG, researchers have developed a wide variety of nanoprobes based on ICG with nanosynthesis strategies.^[^ ^12^ ^]^ However, most of them were “always‐on” probes with relatively high background signals and limited tumor‐to‐background ratio (TBR).^[^ ^13^ ^]^

In contrast, the “turn‐on” nanoprobe holds promise for tumor‐specific imaging because its signal can be switched on only in certain trigger conditions, which dramatically eliminates the background signal in normal tissue.^[^ ^14^ ^]^ Therefore, developing a “turn‐on” nanoprobe with a tumor microenvironment‐activatable feature is significant for precise imaging‐guided surgery. To fabricate an activatable system for “turn‐on” imaging, the achievement of efficient quenching and controllable activation at the tumor site is the key issue.^[^ ^15^ ^]^

**Scheme 1:**
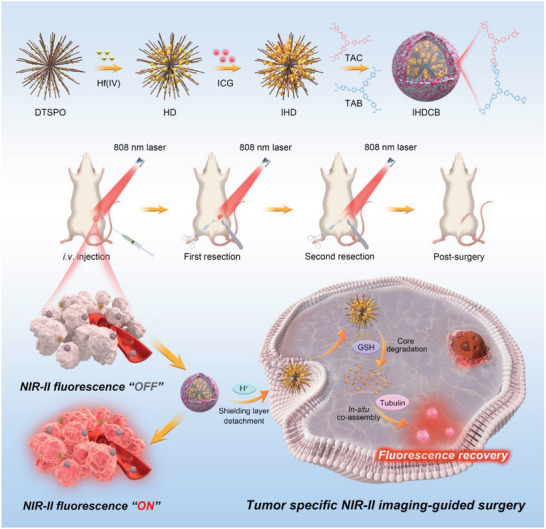
Schematic illustration of a Hf(IV)‐coordinated NIR‐II fluorescence “turn‐on” nanoprobe IHDCB for precise imaging‐guided surgery.

It is worth mentioning that in our preliminary exploration, we unexpectedly discovered that Hf(IV) ions could efficiently quench the fluorescence of ICG by dynamic quenching effect, and this quenching effect is recoverable by co‐assembling with intracellular proteins in vivo. Based on this discovery, for the first time, we developed a Hf(IV)‐coordinated NIR‐II fluorescence “turn‐on” nanoprobe to guide precision surgery (**Scheme** **1**). The nanoprobe was designed by in situ‐coordinated ICG/Hf(IV) within glutathione (GSH)‐responsive dendritic tetra‐sulfide‐bridged porous organosilica, and further enclosed by tumor acidity‐responsive catechol‐borate crosslinking shielding layer. This nanoprobe took full advantage of the tumor microenvironment characteristics (weak acidity and high GSH),^[^ ^16^ ^]^ achieving fluorescence recovery with the stepwise response and showing a remarkable fluorescence “quenching‐recovery‐amplification” process in tumors. The application of this nanoprobe could ultimately realize submillimeter‐sized tumor visualization and precise surgical resection in breast cancer.

## 2 Results

### 2.1 Preparation and Characterization of IHDCB

**Figure 1:**
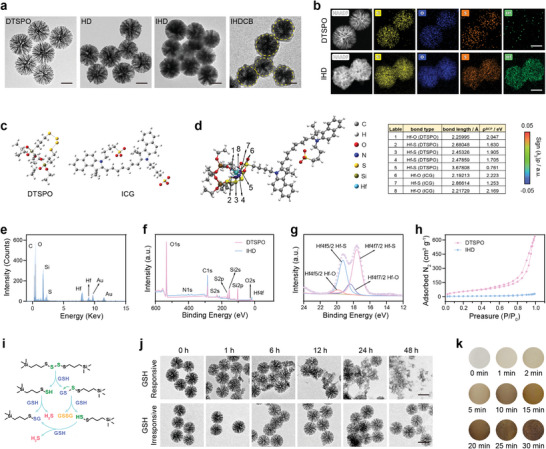
Synthesis and characterization of nanoparticles. a) TEM images of DTSPO, HD, IHD, and IHDCB. Scale bars = 100 nm. b) HAADF image along with element mapping images (Si, O, S, and Hf) of DTSPO and IHD. Scale bars = 100 nm. c) Structures of the tetra‐sulfide motif of DTSPO and ICG. d) IGMH map of IHD. The bond critical points and bond paths corresponding to Hf–O and Hf–S interactions were visible in the IGMH image. Electron density values at the BCP were provided in the table. e) EDS spectra of IHD. f) Full XPS spectra of DTSPO and IHD. g) XPS spectrum in the Hf4f region of IHD. h) N_2_ adsorption/desorption isotherms of DTSPO and IHD. i) Schematic diagram of tetra‐sulfide breakage by GSH and subsequent H_2_S generation. j) TEM images of DTSPO and DPO (free of tetra‐sulfide bridge) immersed in 10 mm of GSH solution for various durations. Scale bars = 200 nm. k) Time‐dependent color change of lead acetate test paper immersed in DTSPO (2 mg mL^−1^) pretreated with 10 mM of GSH solution, indicating the H_2_S generation.

The dendritic tetra‐sulfide‐bridged porous organosilica (denoted as DTSPO) nanoparticle was synthesized using a co‐condensation method.^[^ ^17^ ^]^ Then, various synthesis methods were explored to improve the loading of ICG‐Hf(IV) coordination compounds. However, direct loading of ICG‐Hf(IV) within DTSPO resulted in low efficiency and impurity, as observed through TEM (transmission electron microscopy) images (Figure S1, Supporting Information). In contrast, the utilization of the “controllable space‐limited coordination” approach, which involved the sequential coordination of Hf(IV) ions with DTSPO followed by ICG, resulted in significantly enhanced loading efficiency and purity. Initially, we introduced DTSPO into ethanol and added hafnium chloride (HfCl_4_) under rapid stirring, resulting in the formation of Hf(IV)‐incorporated DTSPO (denoted as HD) nanoparticle through Hf(IV)‐tetra‐sulfide (‐S‐S‐S‐S‐) coordination. Subsequently, we encapsulated ICG into HD (denoted as IHD) by introducing an ethanol solution of ICG into the HD, facilitated by a robust Hf(IV)‐SO_3_^−^ coordination interaction. As shown in **Figure** **1**a, the nanocarrier DTSPO with its radial large porous structures, was nearly fully occupied with ICG‐Hf(IV) by this “controllable space‐limited coordination” strategy, which was confirmed by TEM images. To display the differences between HD and IHD, the enlarged TEM images of HD and IHD, as well as a corresponding quantitative analysis were provided (Figure S2a,b, Supporting Information). The element mapping images of both DTSPO and IHD exhibited a uniform distribution of Si/O/S, and Si/O/S/Hf elements could be detected throughout the entire architecture of IHD. Notably, the signal of Hf in IHD was significantly observed compared to DTSPO, suggesting the successful introduction of Hf within DTSPO (Figure 1b; Figure S3, Supporting Information). Besides, the X‐ray photoelectron spectra (XPS) of the high‐resolution S2p region and the change in the color of the product collected by centrifugation all demonstrated the successful introduction of ICG within HD (Figures S4,S5, Supporting Information). However, the porous structure of DTSPO posed a risk of premature leakage of ICG‐Hf(IV) coordination compounds during blood circulation in vivo. To secure the confinement of dendritic pore channels and avoid the premature leakage induced unwanted fluorescence recovery of ICG‐Hf(IV), the surface of IHD was functionalized with three‐armed boronic acid (TAB, verified by ^1^H NMR spectrum; Figure S6, Supporting Information) and three‐armed catechol (TAC, verified by ^1^H NMR spectrum; Figure S7, Supporting Information) via an interface crosslinking approach. This modification strategy was inspired by polyphenol coatings formed through dynamic covalent interactions between catechol‐rich natural polyphenols and boronic acid groups.^[^ ^18^ ^]^ This dynamic covalent interaction, specifically the phenyl‐borate ester bond, remains stable at neutral pH but undergoes cleavage at weakly acidic pH, which ensures the targeted release and fluorescence recovery of ICG‐Hf(IV) in the tumor microenvironment. The chemical structures of TAB, TAC, and TAC‐TAB crosslinking (denoted as CB) are shown in Figure S8, Supporting Information. TEM images clearly revealed the existence of the CB shielding layer on the surface of IHDCB (Figure 1a). Besides, the B element mapping images of IHDCB further confirmed the successful modification of the CB layer (Figure S9, Supporting Information). Due to the CB layer modification, a slight increase in the hydrodynamic particle size was observed (Figure S10a, Supporting Information). In addition, upon exposure to weak acidity, the IHDCB surface underwent a charge reversal (Figure S10b, Supporting Information), resulting from the weak acidity‐triggered phenylborate‐catechol bond cleavage followed by CB detachment (Figure S11, Supporting Information). Moreover, atomic‐level analysis of DTSPO's tetra‐sulfide motif and ICG molecules within IHD was conducted (Figure 1c). We employed an independent gradient model based on Hirshfeld partition (IGMH) to quantify the interaction strengths of the tetra‐sulfide ligand of DTSPO and sulfonate ligand of ICG with Hf(IV) ions. Subsequently, we employed IGMH analysis alongside the bond critical point (BCP) within the atoms‐in‐molecules (AIM) theory to determine the strengths of the Hf─O bond and Hf─S bonds. The analysis unveiled robust coordinate bonding between DTSPO and ICG with Hf(IV), thereby suggesting a plausible explanation for the significantly elevated loading efficiency (Figure 1d). This conclusion was further corroborated by energy‐dispersive X‐ray spectra (EDS) (Figure 1e) and XPS (Figure 1f,g). Brunauer–Emmett–Teller (BET) analysis demonstrated that it decreased significantly from the original BET surface area of 203.576 (DTSPO) to 17.097 m^2^ g^−1^ (IHD) (Figure 1h). Furthermore, we have generalized this “controllable space‐limited coordination” strategy to 56 elements up until now, preliminarily demonstrating its reliability and broad applicability (Figure S12, Supporting Information).

It is well‐established that the tumor microenvironment exhibits significantly elevated GSH concentration compared to normal tissue.^[^ ^19^ ^]^ It is also reported that the tetra‐sulfide bond could be cleaved by GSH, resulting in the generation of hydrogen sulfide (H_2_S) gas (Figure 1i).^[^ ^17^ ^]^ To assess the GSH responsiveness of DTSPO, we monitored the decomposition of DTSPO (containing tetra‐sulfide linkage) and DPO (without tetra‐sulfide linkage) upon exposure to GSH solution (10 mM). As shown in Figure 1j, the structural distortion of DTSPO was observed at 6 h. As the time increased to 48 h, the morphology of DTSPO distinctly collapsed. In comparison, the DPO almost remained the intact structure at the same time point. In addition, a test paper impregnated with Pb(Ac)_2_ was used to assess the H_2_S generation.^[^ ^20^ ^]^ As shown in Figure 1k, the test strip darkened over time, indicating the H_2_S generation and further confirming the degradation of DTSPO.

### 2.2 NIR‐II Fluorescence Quenching and Recovery In Vitro

**Figure 2:**
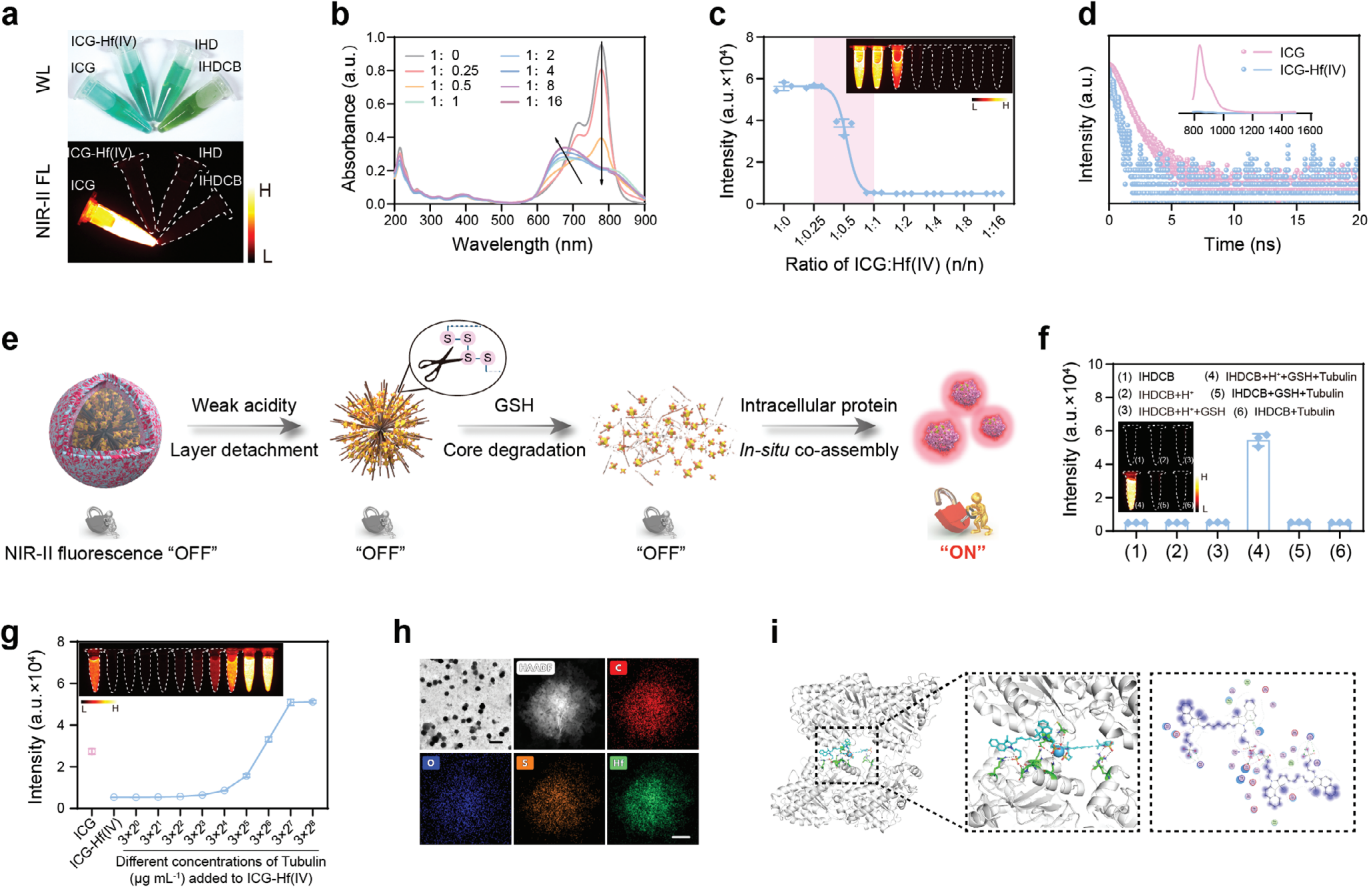
NIR‐II fluorescence quenching and recovery. a) White light and NIR‐II fluorescence images of ICG, ICG‐Hf(IV), IHD, and IHDCB. b) UV–vis–NIR absorption spectra of ICG‐Hf(IV) with different molar ratios. c) Fluorescence intensity of ICG‐Hf(IV) with different molar ratios. d) Time‐resolved fluorescence spectra of ICG and ICG‐Hf(IV). Insert: Fluorescence spectra of ICG and ICG‐Hf(IV). e) Illustration of NIR‐II fluorescence recovery mechanism. f) Fluorescence intensity of IHDCB treated with weak acidity (pH 6.5), GSH, and tubulin. g) Fluorescence intensity of ICG‐Hf(IV) incubated with different concentrations of tubulin. h) TEM images (scale bar = 250 nm), HAADF image along with element mapping images (scale bar = 25 nm) of IHDCB pretreated with weak acidity (pH 6.5) and GSH, and then incubated with tubulin. i) Theoretical simulation of tubulin banding to ICG‐Hf(IV) by gliding docking mode. WL wight light, FL fluorescence.

To investigate the fluorescence quenching and recovery process, a series of explorations were conducted. NIR‐II fluorescence imaging in **Figure** **2**a revealed a significant quenching of ICG by Hf(IV). This quenching effect was unaffected by the introduction of DTSPO and CB layer. The corresponding mean fluorescence intensity (MFI) is shown in Figure S13, Supporting Information. To investigate the specificity of ICG fluorescence quenching by Hf(IV), the fluorescence quenching effects of various metal ions including Ca(II), Bi(III), Gd(III), Lu(III), Yb(III), Cu(II), and Ce(III) were examined. The interaction of metal ions with ICG resulted in varying extents of fluorescence quenching. Notably, Hf(IV) demonstrated a significantly stronger quenching effect on ICG fluorescence compared to others (Figure S14a,b, Supporting Information). Subsequently, with a constant ICG concentration, different ratios of ICG and Hf(IV) were mixed and allowed to coordinate. As the concentration of Hf(IV) increased, a decrease in the characteristic absorption peak of ICG at 780 nm was observed, along with a blue shift in the absorption peak (Figure 2b). Furthermore, within a certain concentration range, Hf(IV) exhibited a concentration‐dependent quenching effect (Figure 2c). The fluorescence emission spectra revealed that the fluorescence intensity of ICG was ≈34.19 times higher than that of the ICG‐Hf(IV) coordination compounds (Figure 2d). Notably, the off‐peak emission spectra of ICG in the NIR‐II region exhibited appreciable quantum yield, which was the basis for ICG‐based NIR‐II imaging (Figure 2d; Figure S15, Supporting Information). In addition, the introduction of DTSPO nanocarrier and modification of the CB layer did not significantly affect the absorption and emission properties of ICG‐Hf(IV) (Figure S16a,b, Supporting Information). To investigate the quenching mechanism, we performed the time‐resolved fluorescence spectroscopy on both ICG and ICG‐Hf(IV). The fluorescence lifetime of ICG‐Hf(IV) was significantly shorter compared to ICG (Figure 2d). This indicated that the presence of Hf(IV) facilitated an energy transfer mechanism during the ICG‐Hf(IV) coordination, resulting in the dynamic quenching of ICG fluorescence.

It could well be imagined that within the tumor microenvironment, the extracellular weak acidity and intracellular overproduced GSH could sequentially trigger the CB shielding layer detachment and IHD core degradation for “release and re‐exposure of ICG‐Hf(IV)” (Figure 2e). To simulate the fluorescence recovery process of nanoprobe in vivo, IHDCB was treated sequentially with weak acidity, GSH, and tubulin. In this study, tubulin was chosen as a model intracellular protein, due to its wide distribution within tumor cells as a cytoskeletal protein.^[^ ^21^ ^]^ As shown in Figure 2f, the fluorescence could be significantly restored when coordinated with tubulin subsequently. To further explore the fluorescence recovery mechanism, we mixed ICG‐Hf(IV) with varying concentrations of tubulin. As the tubulin concentration increased, the fluorescence intensity of ICG‐Hf(IV) gradually recovered and intensified (Figure 2g), which was most likely due to the increase in the fluorescence quantum yield of ICG.^[^ ^22^ ^]^ Additionally, self‐assembly might have occurred between ICG‐Hf(IV) and intracellular protein during this process. To further validate our hypothesis, we pretreated the IHDCB with weak acidity and GSH, followed by incubating it with tubulin for 12 h to assess its state. Using TEM, we observed well‐defined nearly spherical nanoparticles with a diameter of ≈100 nm (Figure 2h). Moreover, the element mappings revealed a uniform distribution of C, O, S, and Hf elements across the nanoparticles (Figure 2h). These results indicated that the released ICG‐Hf(IV) could co‐assemble with intracellular proteins in situ. To better understand the binding mechanism of tubulin with ICG‐Hf(IV), a non‐covalent molecular docking simulation was carried out (Figure 2i; Figure S17, Supporting Information). As shown in Figure 2i, a series of amino acids of tubulin could bind to ICG‐Hf(IV) with multiple interaction forces, which further confirms the above conclusions.

### 2.3 Cytotoxicity and Endocytosis of IHDCB in 4T1 Cells

**Figure 3:**
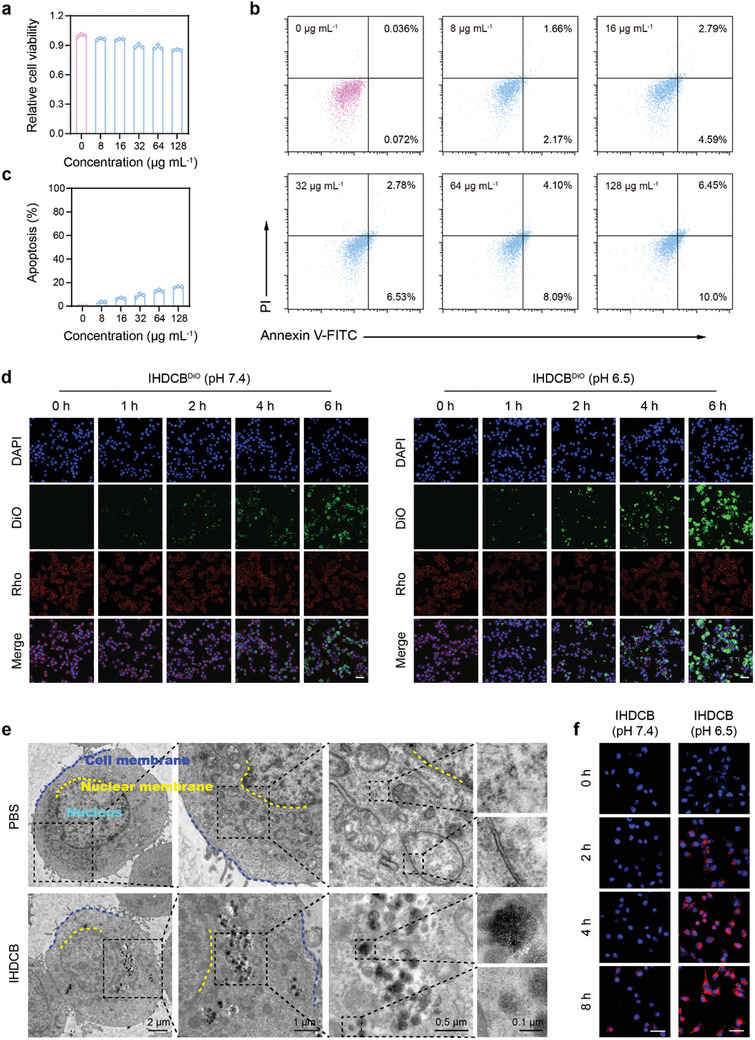
Cytotoxicity, cellular uptake, and fluorescence recovery in 4T1 cells. a) Cytotoxicity of 4T1 cells treated with IHDCB at different concentrations (*n* = 3). b,c) Flow cytometric analysis (b) and relative quantification (c) of the apoptosis in 4T1 cells treated with IHDCB at different concentrations (*n* = 3). d) CLSM images of 4T1 cells after incubation with IHDCB labeled with DiO dye at pH 7.4 and pH 6.5 (*n* = 3). Scale bars = 40 µm. e) Bio‐TEM images of 4T1 cells after incubation with IHDCB at pH 6.5 (*n* = 3). f) Fluorescence microscope images of 4T1 cells at 0, 2, 4, and 8 h post‐incubated with IHDCB at pH 7.4 and pH 6.5 (*n* = 3). Scale bars = 40 µm. Data are presented as mean values ± SD. Rho rhodamine.

Initially, we evaluated the potential cytotoxicity and biosafety of nanoprobes at the cellular level. The cytotoxicity of IHDCB was examined by Cell Counting Kit‐8 (CCK‐8) assay. As shown in **Figure** **3**a, the nanoprobe exhibited almost negligible cytotoxicity toward 4T1 cells. Furthermore, a similar conclusion could be obtained in the apoptosis analysis using the Annexin V‐FITC/PI assay (Figure 3b,c). To investigate the effect of the CB shielding layer on cellular endocytosis, 4T1 cells were incubated with IHDCB labeled with DiO dye at pH 7.4 and pH 6.5. Rhodamine‐labeled Wheat Germ Agglutinin (Rhodamine‐WGA) was used to locate the cell membrane. As shown in Figure 3d, the IHDCB at pH 6.5 exhibited significantly stronger green fluorescence in 4T1 cells compared to the other group at the same time points, which was due to the degradation of the CB shielding layer in weak acidity leading to the surface charge reversal from negative to positive and accelerating cellular endocytosis of nanoprobes. Additionally, to visualize the endocytosis process of nanoprobes, 4T1 cells were incubated with weak acidity pretreated IHDCB for 12 h and examined using bio‐TEM. As shown in Figure 3e, nanoprobes degraded at different rates could be clearly observed inside cells, which further validated the degradation properties of IHDCB in vitro. Afterward, the fluorescence recovery in 4T1 cells was explored. As shown in Figure 3f, the fluorescence intensity of ICG gradually increased over time in 4T1 cells. In addition, at the same time point, the weaker fluorescence intensity was observed in 4T1 cells treated with IHDCB (pH 7.4), which was due to the existence of the CB shielding layer, blocking the premature release of ICG‐Hf(IV) and preventing fluorescence recovery. Compared to the changes between the two groups in Figure 3d (Figure S18, Supporting Information), which was more significant in Figure 3f (Figure S19, Supporting Information). It was because the fluorescence observed in Figure 3d was from the “always on” dye DiO, which changed only due to CB shielding layer degradation, whereas the fluorescence observed in Figure 3f was from quenched ICG, which was not only affected by the CB shielding layer degradation but also processed the fluorescence “quenching‐recovery‐amplification”.

### 2.4 Tumor‐Specific Imaging In Vivo

**Figure 4:**
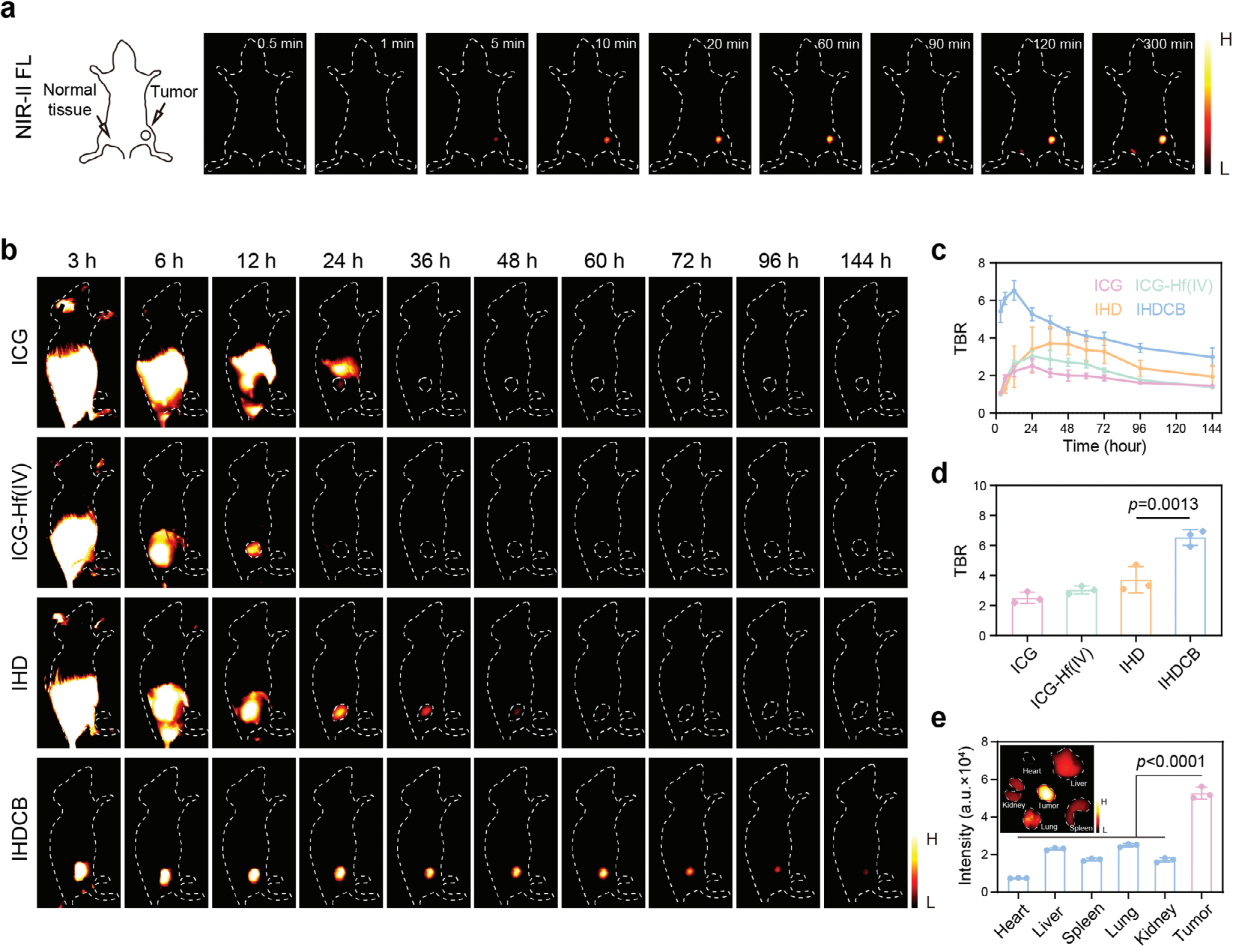
Tumor‐specific imaging in vivo. a) NIR‐II fluorescence images of mice subjected to both subcutaneous injection (left) and intratumoral injection (right) with IHDCB at different time points (*n* = 3 mice). b–d) NIR‐II fluorescence images (b) and corresponding quantitative analysis (c,d) of TBR of mice intravenously injected with ICG, ICG‐Hf(IV), IHD, and IHDCB (*n* = 3 mice). e) NIR‐II fluorescence images and corresponding fluorescence intensity of major organs (heart, liver, spleen, lung, and kidney) and tumors from mice injected with IHDCB. Data are presented as mean values ± SD. Statistical significance was calculated via one‐way ANOVA with Tukey's multiple comparisons test (d,e).

To evaluate the sensitivity of IHDCB toward tumor tissue in vivo, identical quantities of IHDCB were administered both subcutaneously and intratumorally to 4T1 tumor‐bearing mice. As shown in **Figure** **4**a, a remarkable enhancement in fluorescence signal was observed in the tumor site, whereas minimal changes were detected in the normal tissue during the same period. To further validate the tumor‐specific imaging abilities of IHDCB, 4T1 tumor‐bearing mice were intravenously injected with various formulations including ICG, ICG‐Hf(IV), IHD, and IHDCB. As shown in Figure 4b, the IHDCB group showed a clearly defined and intense fluorescence signal at the tumor sites. Furthermore, the fluorescence signal intensity could persist for up to 144 h post‐injection. Notably, the maximum TBR in the IHDCB group was significantly higher than others (Figure 4c,d). In addition, NIR‐II fluorescence imaging was conducted on dissected mouse tissue, including heart, liver, spleen, lung, kidney, and tumor, revealing that the tumor tissue exhibited significantly stronger fluorescence signal (Figure 4e). This observation further underscored the remarkable tumor‐specific imaging capability of IHDCB.

### 2.5 Submillimeter‐Sized Tumors Identification In Vivo

**Figure 5:**
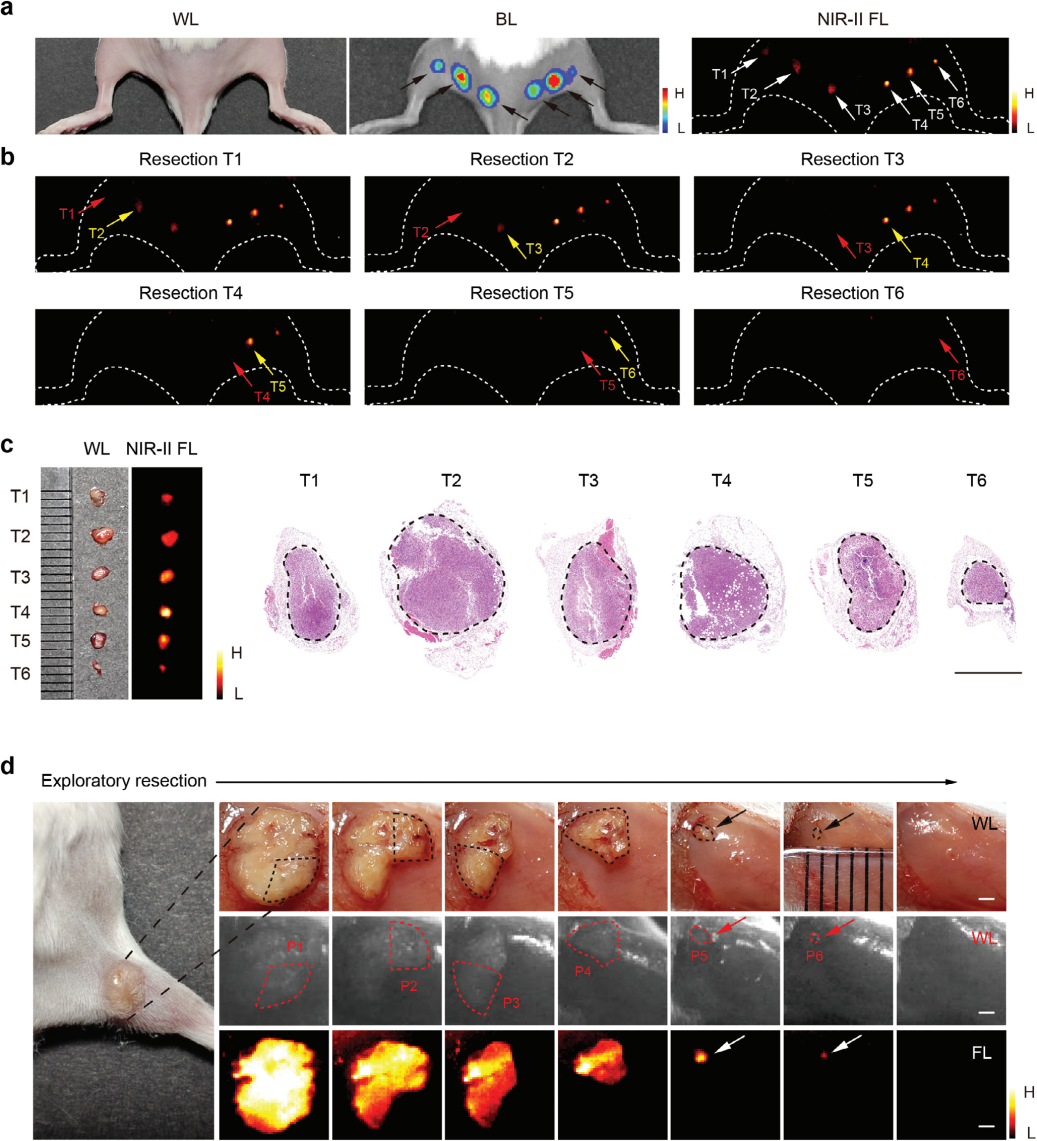
Submillimeter‐sized tumor identification in vivo. a) White light, bioluminescence, and NIR‐II fluorescence images of multiple‐microtumor mouse model injected with IHDCB (*n* = 3 mice). b) The process of microtumor excision with the guidance of NIR‐II fluorescence. c) White light, NIR‐II fluorescence, and corresponding H&E staining images of resected microtumor tissue. Scale bar = 1 mm. d) White light and NIR‐II fluorescence images of exploratory resection experiment for minimal identifiable residual lesions (*n* = 3 mice). Scale bars = 1 mm. BL bioluminescence, T tissue, P piece.

To evaluate the ability of IHDCB to recognize primary microtumors, the mouse model bearing multiple microtumors was established by subcutaneous injection of a limited number of 4T1‐Luc cells at various locations on the mouse back. The small size of the tumors posed challenges for recognition under white light. However, six micro‐lesions were visible on the fluorescence images, exhibiting high consistency with the bioluminescence result (**Figure** **5**a). Afterward, six microtumors were successively excised by the NIR‐II fluorescence guidance (Figure 5b). The corresponding H&E staining images revealed the diameter of six microtumors, ranging between ≈0.5–2 mm. Notably, T6 with a diameter less than 1 mm could still be accurately identified, indicating that this ultrasensitive nanoprobe was feasible for precise visualization of submillimeter‐sized tiny tumors (Figure 5c). Additionally, the entire borderline of each microtumor was effectively distinguished from adjacent normal tissue (Figure 5c).

To evaluate the capability of residual microtumor identification, we conducted an exploratory, multi‐step resection on a subcutaneous tumor. As shown in Figure 5d, the IHDCB‐based NIR‐II fluorescence imaging could clearly identify tiny residual tumors smaller than 1 mm. The demonstrated ability to accurately identify primary and residual submillimeter‐sized tumors served as the basis for subsequent IHDCB‐based fluorescence imaging‐guided surgery.

### 2.6 Imaging‐Guided Subcutaneous 4T1‐Luc Tumor Resection

**Figure 6:**
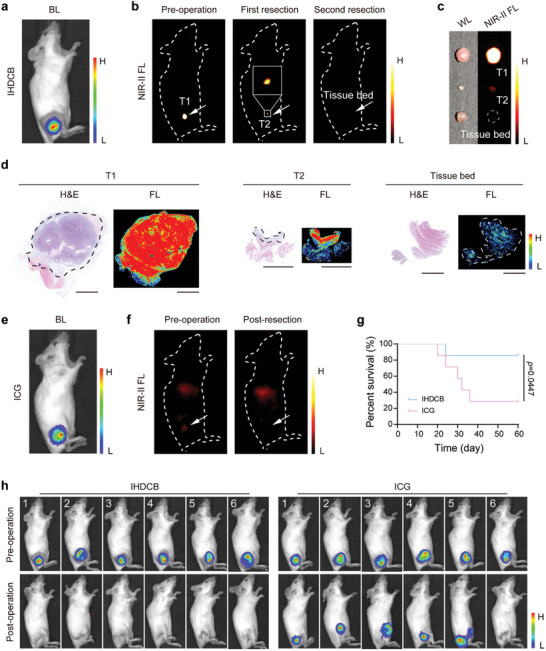
NIR‐II fluorescence imaging‐guided surgery in mice bearing subcutaneous 4T1‐Luc tumor. a–c) NIR‐II fluorescence imaging‐guided surgery in the subcutaneous tumor‐bearing mice injected with IHDCB. Preoperative bioluminescence image (a), intraoperative NIR‐II fluorescence images (b), as well as white light and NIR‐II fluorescence images of resected tissue (c) of subcutaneous tumor‐bearing mice. d) H&E staining images and corresponding fluorescence images of resected tissue slices (4 µm) in Figure 6c. Scale bars = 2 mm. e, f) NIR‐II fluorescence imaging‐guided surgery in the subcutaneous tumor‐bearing mice injected with ICG. Preoperative bioluminescence image (e) and intraoperative NIR‐II fluorescence images (f) of subcutaneous tumor‐bearing mice. g) Survival analysis of mice in different groups. h) Bioluminescence images of the subcutaneous tumor‐bearing mice pre‐operation (up) and 14 days post‐operation (down) under IHDCB‐based NIR‐II fluorescence imaging‐guided surgery (*n* = 6 mice). Statistical significance was calculated via log‐rank (Mantel–Cox) test (g).

Inspired by the remarkable tumor recognition capability of IHDCB, we employed NIR‐II fluorescence imaging to aid surgical navigation in the mice bearing subcutaneous 4T1‐Luc tumor in vivo. Initially, we utilized bioluminescence imaging to visualize the tumor location (**Figure** **6**a). Then, the preoperative NIR‐II fluorescence imaging was carried out to precisely identify the tumor location. After the first resection under white light, fluorescence imaging was employed to pinpoint any potential tumor residues, which were difficult to discern with the naked eyes. This approach facilitated subsequent imaging‐guided precise resection. Once the complete removal of the tumor was confirmed by the absence of a fluorescence signal, the wound was sutured accordingly (Figure 6b,c). The corresponding surgical procedure under white light is shown in Figure S20, Supporting Information. Afterward, H&E staining and fluorescence signal scanning were performed on the excised tissues. As shown in Figure 6d, the tumor area visualized by H&E staining closely corresponded to the area exhibiting a strong NIR‐II fluorescence signal, which indicated the excellent tumor recognition ability of IHDCB. The negative tissue beds further indicated the complete resection of tumor lesions. Similarly, tumor resection was conducted under ICG imaging guidance (Figure 6e,f). Furthermore, the tumor recurrence and survival of the mice were monitored. The NIR‐II fluorescence imaging‐guided surgery, utilizing IHDCB, achieved the most complete tumor resection and significantly reduced local recurrence rate and improved the overall survival rate of mice compared to the ICG group (Figure 6g,h). Through the course of this experiment, the body weight of the mice slightly decreased after surgery, which was due to anesthesia and trauma induced by tumor resection affecting the feeding of mice. Afterward, the body weight of the mice gradually increased over time (Figure S21, Supporting Information).

### 2.7 Imaging‐Guided Muscle‐Infiltrating 4T1‐Luc Tumor Resection

**Figure 7:**
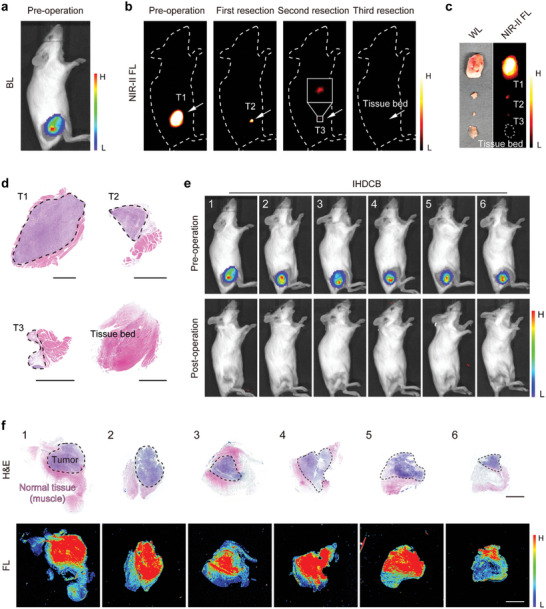
NIR‐II fluorescence imaging‐guided surgery in mice bearing muscle‐infiltrating 4T1‐Luc tumor. a,b) Preoperative bioluminescence images (a) and intraoperative NIR‐II fluorescence images (b) of muscle‐infiltrating tumor‐bearing mice. c, d) White light, NIR‐II fluorescence images (c), and corresponding H&E staining images (scale bars = 2 mm) (d) of resected tissue. e) Bioluminescence images of the muscle‐infiltrating tumor‐bearing mice pre‐operation (up) and 14 days post‐operation (down) under IHDCB‐based NIR‐II fluorescence imaging‐guided surgery (*n* = 6 mice). f) H&E staining images and corresponding fluorescence images of resected tissue slices (4 µm). Scale bars = 2 mm.

Though the subcutaneous tumor model is an idealized animal model, a proportion of breast tumors are invasive.^[^ ^23^ ^]^ To simulate this situation, a mouse model bearing a muscle‐infiltrating 4T1‐Luc tumor was constructed. Similar to the procedure of imaging‐guided subcutaneous tumor resection, we utilized bioluminescence imaging to visualize the tumor location first (**Figure** **7**a). Afterward, IHDCB was injected through the tail vein and the surgery was performed. NIR‐II imaging accurately revealed the tumor location, which was consistent with the bioluminescence results. Subsequently, IHDCB‐based imaging‐guided stepwise resection of the muscle‐infiltrating tumor was performed (Figure 7b). White light, fluorescence imaging, as well as H&E staining of excised tissue were performed. In comparison to the tissue bed, T1, T2, and T3 exhibited significantly stronger fluorescence signals (Figure 7c). H&E staining results further confirmed that T1, T2, and T3 contained tumor tissue, and the tissue bed did not contain tumor cells, indicating the complete surgical resection (Figure 7d). On day 14 following surgery, bioluminescence imaging results showed no recurrence (Figure 7e). During this period, the body weight of the mice slowly increased after a slight decrease caused by surgery (Figure S22, Supporting Information). To further investigate its tumor recognition ability at the microscopic level, we randomly excised the muscle‐infiltrating tumor tissue from another six mice injected with IHDCB. As shown in Figure 7f, the tumor area visualized by H&E staining closely corresponded to the area exhibiting a strong NIR‐II fluorescence signal. In contrast, the surrounding muscle tissue exhibited a significant weaker fluorescence signal (Figure S23, Supporting Information). It reinforced the exceptional tumor recognition capability of IHDCB.

### 2.8 Imaging‐Guided Orthotopic 4T1‐Luc Tumor Resection

**Figure 8:**
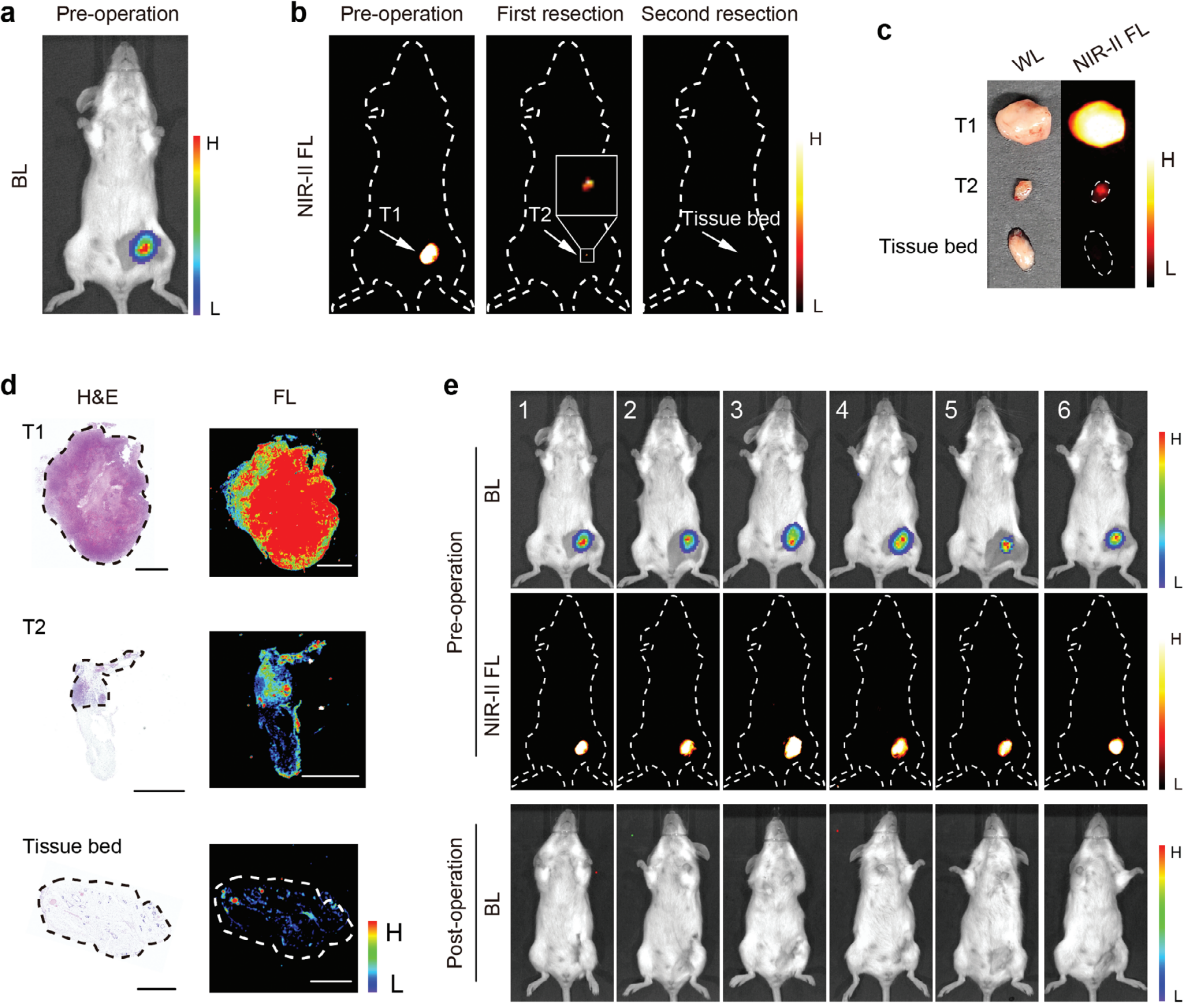
NIR‐II fluorescence imaging‐guided surgery in mice bearing orthotopic 4T1‐Luc tumor. a,b) Preoperative bioluminescence images (a) and intraoperative NIR‐II fluorescence images (b) of orthotopic tumor‐bearing mice. c) White light and NIR‐II fluorescence images of resected tissue. d) H&E staining images and corresponding fluorescence images of resected tissue slices (4 µm) in Figure 8c. Scale bars = 2 mm. e) Bioluminescence images and NIR‐II fluorescence images of mice bearing orthotopic tumor pre‐operation (up) and 14 days post‐operation (down) under IHDCB‐based NIR‐II fluorescence imaging‐guided surgery (*n* = 6 mice).

To further mimic the resection of breast cancer in clinical practice, we established a mouse model bearing an orthotopic 4T1‐Luc tumor. The bioluminescence imaging indicated that the tumor was located in the fourth pair of mammary glands on the mouse's left side (**Figure** **8**a). Next, fluorescence imaging was conducted to pinpoint any tumor residue and guide the precise resection until complete removal was achieved. After the second resection, no signal was detected at the original tumor site, indicating successful and complete tumor removal (Figure 8b). Afterward, the wound was sutured accordingly. The corresponding surgical images under white light are shown in Figure S24, Supporting Information. In comparison to the tissue bed, tumor tissue exhibited a significantly stronger fluorescence signal (Figure 8c). In addition, histological examination using H&E staining revealed that the T2 adhered to a small part of the normal tissue, and no tumor cells were detected in the tissue adjacent to the resection margin (Figure 8d). Furthermore, the significant NIR‐II fluorescence signal area in the tissue was highly consistent with the tumor area visualized through H&E staining (Figure 8d). Figure 8e displayed the preoperative and postoperative bioluminescence and fluorescence imaging of six mice bearing orthotopic tumors, undergoing IHDCB‐based surgical navigation. The results indicated that all six mice exhibited no signs of recurrence 14 days post‐surgery, suggesting a precise and comprehensive surgical resection was demonstrated. Besides, the body weight curve of mice showed a slight decrease after the operation, but the change was within 7% and the body weight increased afterward with wound healing and normal feeding (Figure S25, Supporting Information).

### 2.9 Biosafety Evaluation

To evaluate the biosafety of IHDCB, the blood routine and blood biochemical variables tests were performed on days 0, 1, 3, 7, and 28 after intravenous injection of IHDCB into mice. As shown in Figure S26a, Supporting Information, all values remained within the normal range. In addition, histopathological examination of major organs (heart, liver, spleen, lung, and kidney) of mice validated the absence of significant toxicity from IHDCB (Figure S26b, Supporting Information). Furthermore, the body weight changes of mice were also monitored after intravenous injection of IHDCB, and no noteworthy decrease was observed (Figure S26c, Supporting Information). Moreover, in the hemolysis assay, IHDCB did not exhibit significant hemolysis when incubated with red blood cells for 3 h, suggestive of its low blood toxicity (Figure S26d, Supporting Information). These results confirmed the excellent biocompatibility of IHDCB, providing potential biosafety assurance for future clinical applications.

## 3 Discussion

For early‐stage breast cancer patients, precise intraoperative localization and complete excision of residual lesions are crucial for the successful implementation of BCS with reduced postoperative recurrence.^[^ ^24^ ^]^ The NIR fluorescent dye ICG has been approved for clinical use by the Food and Drug Administration (FDA), and the exhibited off‐peak emission spectra in the NIR‐II region have led to extensive research.^[^ ^25^ ^]^ However, the poor tumor enrichment efficiency of ICG limits its application in accurate tumor resection. To solve this problem, our group has developed a series of nanoprobes based on this traditional dye. Two types of rare‐earth oxide nanoprobes cRGD‐targeted hollow gadolinium oxide nanoparticles loaded with ICG (R&HV‐Gd@ICG)^[^ ^26^ ^]^ and folate‐targeted silica oxide‐gadolinium oxide core–shell nanoparticle loaded with ICG (VGd@ICG‐FA),^[^ ^27^ ^]^ as well as one self‐assembled nanoprobe gadolinium‐diethylenetriaminepentaacetic acid‐human serum albumin nanoparticle co‐loaded with ICG and bevacizumab (^Gd^DTPA‐HSA@ICG‐Bevacizumab)^[^ ^10^ ^]^ were developed for fluorescence imaging‐guided surgery and enhanced radiotherapy in breast cancer. However, all of them were “always‐on” probes, resulting in relatively high background signals and limited specificity for tumors.

Interestingly, in our preliminary exploration, we unexpectedly discovered that lanthanum metal Hf(IV) ions could efficiently quench the fluorescence of ICG, and this quenching effect is recoverable in vivo. In addition, we investigated the mechanism of fluorescence quenching and recovery by delving into the structure of the material. Based on this discovery, we developed a Hf(IV)‐coordinated NIR‐II fluorescence “turn‐on” nanoprobe IHDCB. This activatable nanoprobe was constructed by co‐coordinated ICG and Hf(IV) within DTSPO nanocarrier by “controllable space‐limited coordination” strategy and afterward enclosed by acidity‐sensitive catechol‐borate crosslinking. Within tumor sites, the IHDCB would sequentially respond to extracellular acidity and intracellular overproduced GSH to release ICG‐Hf(IV) coordination. Then the ICG‐Hf(IV) could in situ‐assemble with intracellular protein to recover and amplify the fluorescence of ICG, showing a remarkable fluorescence “quenching‐recovery‐amplification” process.

By utilizing this nanoprobe, we could accurately identify the submillimeter‐sized primary and residual tumors. Inspired by the remarkable tumor recognition capability of IHDCB, surgical navigation experiments were carried out in multiple breast cancer mouse models including subcutaneous, muscle‐infiltrating, and orthotopic breast cancer models. It has repeatedly verified that IHDCB‐based NIR‐II fluorescence imaging‐guided surgery could accurately identify tumor margins, significantly reduce the risk of postoperative recurrence, and improve survival rates. Furthermore, postoperative tissue analysis showed that the fluorescence imaging results were highly consistent with tissue H&E staining results, which further verified the precise identification of tumors by this ultrasensitive nanoprobe.

For inorganic nanoprobes, especially those containing heavy metals, biosafety issues are crucial for further clinical applications. In our study, the heavy metal element Hf was introduced. We have taken biosafety issues into account seriously, for which in vitro and in vivo experiments were conducted and all the results demonstrated that the IHDCB nanoprobe showed excellent biocompatibility. In addition, it is worth mentioning that a phase 2–3 clinical trial in 2019 evaluated the safety and efficacy of the hafnium oxide (HfO_2_) nanoparticle NBTXR3 for radiosensitization, which also verified the feasibility and biosafety of the clinical application of Hf‐introduced nanoprobe.^[^ ^28^ ^]^

In summary, we ingeniously developed an activatable “turn‐on” nanoprobe IHDCB for precise imaging‐guided surgery. Besides, the construction of IHDCB provides a new strategy for ICG‐based responsive nanoprobe design. Although the study mainly focused on breast cancer currently, this nanoprobe is also promising for being generalized to other types of tumors. In addition, considering the relatively complex structure and the lack of in‐depth systemic pharmacokinetic studies, optimization, and investigations are necessary for future translation into clinical practice.

## 4 Experimental Section

### Materials

All of the chemicals were used without further purification. Triethanolamine (TEA) was purchased from Innochem (Beijing, China). Cetyltrimethylammonium bromide (CTAB) was purchased from Sinopharm Chemical Reagent Co., Ltd. (Shanghai, China). Sodium salicylate (NaSal), tetraethyl orthosilicate (TEOS), and bis[3‐(triethoxysilyl)propyl]tetra‐sulfide (BTES) were obtained from Aladdin Reagent, Ltd (Shanghai, China). HfCl_4_ and ICG were purchased from Sigma‐Aldrich (USA). RPMI 1640, fetal bovine serum (FBS), and 0.25% trypsin‐EDTA were purchased from Thermo Fisher Scientific (Shanghai, China). CCK‐8 kit and Annexin V FITC/PI Kit were purchased from LABLEAD (Xiamen, China). Rhodamine‐WGA was purchased from Shanghai Maokang Biotechnology Co., Ltd. (Shanghai, China). *D*‐Luciferin potassium salt was purchased from Beyotime (Shanghai, China). All other reagents were analytical grade and used as received.

### Synthesis of TAC

1, 3, 5‐tris(4‐aminophenyl)benzene (0.5 mmol, 0.176 g) was dissolved in a mixed solvent containing anhydrous ethanol (8 mL) and dichloromethane (4 mL). Then, 3, 4‐dihydroxybenzaldehyde (1.6 mmol, 0.221 g) dissolved in anhydrous ethanol (3 mL) was added to the aforementioned mixture. Subsequently, the reaction mixture was stirred for 12 h under an argon atmosphere and then concentrated to ≈5 mL. The resulting orange precipitate was collected by filtration, washed with cold ethanol, and then dried under a vacuum.

### Synthesis of TAB

1, 3, 5‐tris(4‐aminophenyl)benzene (0.5 mmol, 0.176 g) was dissolved in a mixed solvent containing anhydrous ethanol (16 mL) and dichloromethane (8 mL). Then, 4‐formylphenylboronic acid (1.6 mmol, 0.239 g) dissolved in anhydrous ethanol (3 mL) was added to the aforementioned mixture. Subsequently, the reaction mixture was stirred for 12 h under an argon atmosphere and then concentrated to ≈2.5 mL. Afterward, dichloromethane (12.5 mL) was added into the solution slowly in an ice‐water bath. The resulting yellow precipitate was collected by filtration, washed with a cold mixed solvent containing dichloromethane and ethanol (volume ratio: 5:1), and then dried under a vacuum.

### Synthesis of DTSPO

TEA (60 µL) was added to 25 mL of water and stirred continuously at 80 °C for 0.5 h. Then, 380 mg of CTAB and 150 mg of NaSal were added while stirring was maintained for 2 h. Subsequently, a mixture of TEOS (2 mL) and BTES (1.6 mL) was added dropwise and stirred continuously for 12 h. The product was isolated through centrifugation, washed with ethanol, thoroughly rinsed with HCl (2 m)‐methanol solution, and redispersed in ethanol.

### Synthesis of IHD by “Controllable Space‐Limited Coordination” Strategy

DTSPO (10 mg) was dispersed in 2 mL of ethanol followed by stirring until a homogeneous dispersion was achieved. Then, 6.4 mg of HfCl_4_ dissolved in 2 mL of ethanol was added to the dispersion of DTSPO, and stirring was continued for 12 h. Afterward, 7.8 mg of ICG dispersed in 8 mL of ethanol was added to the above mixture, and stirring was continued in the dark for 1 h. The product was collected by centrifugation and washed with ethanol three times.

### Synthesis of IHD by “Direct Loading” Strategy

Briefly, this method involves synthesizing the ICG‐Hf(IV) coordination and then loading the coordination into DTSPO nanocarriers. First, 6.4 mg of HfCl_4_ dissolved in 2 mL of ethanol and 7.8 mg of ICG dispersed in 8 mL of ethanol were mixed and stirred continuously in the dark for 12 h. Then, 10 mg of DTSPO dispersed in 2 mL of ethanol was added to the above mixture, and stirring was continued in the dark for 6 h. The product was collected by centrifugation and washed with ethanol three times.

### Synthesis of IHDCB

TAC (4 mg) dispersed in 2 mL of methanol was added to the ethanol dispersion of IHD. Subsequently, ultrasonication was applied for 30 s, followed by stirring for 10 min. TAB (4 mg) dispersed in 2 mL of ethanol was added dropwise to the aforementioned mixture and stirring was continued for 30 min. The product was collected by centrifugation and washed with ethanol three times.

### Characterization

TEM images were acquired using a JEM‐2100F transmission electron microscope (Japan). Energy‐dispersive X‐ray element mappings were obtained using a Talos F200 field emission transmission electron microscopy (USA). Hydrodynamic sizes and zeta potentials were recorded on the dynamic light scattering (USA). XPS was obtained on a Thermo Scientific K‐Alpha+ with Al‐Kα source (USA). N_2_ adsorption–desorption isotherm was measured with a Tristar 3000 system (USA). UV–vis–NIR spectra were measured by a Cary 5000 UV–vis–NIR spectrophotometer (USA). Fluorescence emission spectra and fluorescence lifetime were obtained by an Edinburgh FLS980 fluorescence spectrophotometer (UK). NIR‐II fluorescence imaging was performed using a Series III 900/1700 NIR‐II fluorescence imaging system (China).

### In Vitro Degradation

The degradation behavior of DTSPO was tested by detecting the morphology change and H_2_S generation. Briefly, the DTSPO was dispersed in 10 mm of GSH solution and then observed at various time points by using TEM. Furthermore, the H_2_S gas generated by DTSPO degradation was detected by the Pb(Ac)_2_ test paper.

### Cell Culture

The murine breast cancer cell line 4T1 was purchased from the American Type Culture Collection (USA). The 4T1‐Luc cell line was obtained from the Shanghai Zhong Qiao Xin Zhou Biotechnology Co., Ltd. (China). Both 4T1 and 4T1‐Luc cells were cultured in RPMI‐1640 medium, which was supplemented with 10% FBS and 1% penicillin/streptomycin. This culturing process occurred within a standard, humidified cell culture incubator, maintained at 37 °C and 5% CO_2_.

### Cellular Uptake

For DiO labeling, 2 mg of DiO and 10 mg of IHD were dispersed in 2 mL of ethanol, and the mixture was stirred in the dark for 12 h. Subsequently, the product was collected by centrifugation and washed with ethanol three times. Then, the CB crosslinking layer was modified onto the surface to synthesize DiO‐labeled IHDCB. The loading content of DiO was determined to be ≈1.63% through UV–vis–NIR absorption spectroscopy. Furthermore, a dialysis method was employed to assess the cumulative release profiles of both free DiO and DiO‐labeled IHDCB. The cumulative release of DiO from nanoprobes was less than 5% at pH 7.4 over 24 h, indicative of minimal burst release (Figure S27, Supporting Information). It suggested the reliability of DiO labeling for cellular experiments. Afterward, 4T1 cells were seeded into 12‐well plates and cultured overnight. Then the cells were incubated with DiO‐labeled IHDCB (64 µg mL^−1^) at pH 7.4 and 6.5 for different durations. Afterward, the cells were washed with cold PBS and fixed with 4% paraformaldehyde. After washing with cold PBS, the cells were stained with DAPI for 15 min. Finally, images of the cells were captured by confocal laser scanning microscopy (CLSM).

### Cytotoxicity and Apoptosis Analysis

4T1 cells were incubated with a medium containing different concentrations of IHDCB at pH 6.5 for 24 h. Then, the medium was discarded, and the CCK‐8 assay was assessed according to the manufacturer's recommended protocols. For apoptosis analysis, after the medium was discarded, the cells were collected using trypsin, washed with PBS, resuspended in Annexin‐binding buffer, stained with Annexin V‐FITC/PI following the instructions, and analyzed by flow cytometry.

### Animal Preparation

All animal experiments were approved by the Institutional Animal Care and Use Committee of Xiamen University (No. XMULAC20210147) and conducted in strict accordance with the relevant guidelines. Female BALB/c mice (6–8 weeks) were purchased from Guangdong GemPharmatech Co., Ltd. (China).

For the establishment of a mouse model bearing multiple‐microtumors, 10–20 µL 4T1‐Luc cells (7 × 10^6^ mL^−1^) were injected in multiple places on the back of the mouse. After 5 days, NIR‐II fluorescence imaging was carried out. For the establishment of a mouse model bearing subcutaneous tumor, 100 µL 4T1‐Luc cells (7 × 10^6^ mL^−1^) were injected into the right hind flanks of the mice. For the establishment of a mouse model bearing muscle‐infiltrating tumor, 100 µL 4T1‐Luc cells (7 × 10^6^ mL^−1^) were injected into the muscle of the right hind flanks of the mice. For the establishment of an orthotopic breast cancer model, 100 µL 4T1‐Luc cells (7 × 10^6^ mL^−1^) were injected into the fat pad on the fourth pair of mammary glands on the left side of the mice. NIR‐II fluorescence imaging was carried out after the tumor size reached ≈200 mm^3^.

### In Vivo Fluorescence Imaging

For bioluminescence imaging, *D*‐Luciferin potassium salt was dissolved in sterile D‐PBS (without Mg^2+^ and Ca^2+^) to prepare a 15 mg mL^−1^ *D*‐Luciferin potassium salt solution. Then, the prepared *D*‐fluorescent potassium salt solution (10 µL g^−1^) was intraperitoneally injected into 4T1‐Luc tumor‐bearing mice. After 10–20 min, bioluminescence imaging was performed on an IVIS Lumina imaging system (USA). Unless otherwise specified, mice bearing 4T1‐Luc tumors for NIR‐II fluorescence imaging were intravenously injected with IHDCB (5 mg Kg^−1^), and NIR‐II fluorescence imaging was performed using a Series III 900/1700 NIR‐II fluorescence imaging system (China).

### Histological Analysis

After treatment, the mice were euthanized, and the tumors were dissected. Tumor tissue was fixed in 4% paraformaldehyde and embedded in paraffin. Afterward, they were cut into 4 µm tissue sections for subsequent H&E staining and fluorescence signal analysis. The tissue's fluorescence signal was obtained using an Odyssey CLx (USA).

### In Vivo Biosafety

The healthy female BALB/c mice (6–8 weeks) were intravenously injected with IHDCB (10 mg Kg^−1^). On days 0, 1, 3, 7, and 28, the mice were executed. Then, the heart, liver, spleen, lung, and kidney were excised, fixed in 4% paraformaldehyde, embedded in paraffin, and cut into 4 µm of tissue sections for H&E staining. In addition, the blood samples were collected on days 1, 3, 7, and 28 for biochemical and hematological tests.

### Statistical Analysis

Data were collected from at least three independent measurements (*n* ≥ 3). All data were presented as mean values ± SD. Statistically significant differences were analyzed by the two‐tailed unpaired *t‐*test, two‐way ANOVA with Sidak's multiple comparisons test, and one‐way ANOVA with Tukey's multiple comparisons test. The survival benefit was determined using a log‐rank (Mantel–Cox) test. *p‐*values were calculated using GraphPad Prism 8.0.2. *p* < 0.05 was considered significant statistically.

## Conflict of Interest

The authors declare no conflict of interest.

## Supporting information



Supporting Information

## Data Availability

The data that support the findings of this study are available from the corresponding author upon reasonable request.

## References

[1] J. de Boniface , J. Frisell , L. Bergkvist , Y. Andersson , Br. J. Surg. 2018, 105, 1607. 29926900 10.1002/bjs.10889 PMC6220856

[2] L. Wyld , R. A. Audisio , G. J. Poston , Nat. Rev. Clin. Oncol. 2015, 12, 115. 25384943 10.1038/nrclinonc.2014.191

[3] T. Nowikiewicz , E. Śrutek , I. Głowacka‐Mrotek , M. Tarkowska , A. Żyromska , W. Zegarski , Sci. Rep. 2019, 9, 13441. 31530867 10.1038/s41598-019-49951-y PMC6748937

[4] E. L. Rosenthal , J. M. Warram , E. de Boer , J. P. Basilion , M. A. Biel , M. Bogyo , M. Bouvet , B. E. Brigman , Y. L. Colson , S. R. DeMeester , G. C. Gurtner , T. Ishizawa , P. M. Jacobs , S. Keereweer , J. C. Liao , Q. T. Nguyen , J. M. Olson , K. D. Paulsen , D. Rieves , B. D. Sumer , M. F. Tweedle , A. L. Vahrmeijer , J. P. Weichert , B. C. Wilson , M. R. Zenn , K. R. Zinn , G. M. Van Dam , J. Nucl. Med. 2016, 57, 144. 26449839 10.2967/jnumed.115.158915 PMC4772735

[5] M. Goto , I. Ryoo , S. Naffouje , S. Mander , K. Christov , J. Wang , A. Green , A. Shilkaitis , T. K. Das Gupta , T. Yamada , EBioMedicine 2022, 76, 103850. 35108666 10.1016/j.ebiom.2022.103850 PMC8814381

[6] a) F. Wang , Y. Zhong , O. Bruns , Y. Liang , H. Dai , Nat. Photonics 2024, 18, 535;

[7] R. Yang , K. Lou , P. Wang , Y. Gao , Y. Zhang , M. Chen , W. Huang , G. Zhang , Small Methods 2021, 5, 2001066. 10.1002/smtd.202001066 34927825

[8] S. Zhu , B. C. Yung , S. Chandra , G. Niu , A. L. Antaris , X. Chen , Theranostics 2018, 8, 4141. 30128042 10.7150/thno.27995 PMC6096392

[9] S. Zhu , R. Tian , A. L. Antaris , X. Chen , H. Dai , Adv. Mater. 2019, 31, 1900321. 10.1002/adma.201900321 PMC6555689 31025403

[10] Y. Zhang , W. Liu , X. Luo , J. Shi , Y. Zeng , W. Chen , W. Huang , Y. Zhu , W. Gao , R. Li , Z. Ming , L. Zhang , R. Yang , J. Wang , G. Zhang , Adv. Sci. 2023, 10, 2205294. 10.1002/advs.202205294 PMC10074043 36721054

[11] P. Lin , J. Shi , Y. Lin , Q. Zhang , K. Yu , L. Liu , L. Song , Y. Kang , M. Hong , Y. Zhang , Adv. Sci. 2023, 10, 2207486. 10.1002/advs.202207486 PMC10288272 37088829

[12] W. C. W. Chan , BME Front 2023, 4, 0016. 37849661 10.34133/bmef.0016 PMC10085247

[13] Y. Tang , Y. Li , X. Hu , H. Zhao , Y. Ji , L. Chen , W. Hu , W. Zhang , X. Li , X. Lu , W. Huang , Q. Fan , Adv. Mater. 2018, 30, 1801140. 10.1002/adma.201801140 29920793

[14] C. Chen , R. Tian , Y. Zeng , C. Chu , G. Liu , Bioconjugate Chem. 2020, 31, 276. 10.1021/acs.bioconjchem.9b00734 31935072

[15] a) H. Meng , X. Zhang , C. Yang , H. Kuai , G. Mao , L. Gong , W. Zhang , S. Feng , J. Chang , Anal. Chem. 2016, 88, 6057; 27161421 10.1021/acs.analchem.6b01352

[16] R. Song , T. Li , J. Ye , F. Sun , B. Hou , M. Saeed , J. Gao , Y. Wang , Q. Zhu , Z. Xu , H. Yu , Adv. Mater. 2021, 33, 2101155. 10.1002/adma.202101155 34170581

[17] B. Liu , S. Liang , Z. Wang , Q. Sun , F. He , S. Gai , P. Yang , Z. Cheng , J. Lin , Adv. Mater. 2021, 33, 2101223. 10.1002/adma.202101223 34145652

[18] X. Guo , W. Luo , L. Wu , L. Zhang , Y. Chen , T. Li , H. Li , W. Zhang , Y. Liu , J. Zheng , Y. Wang , Adv. Sci. 2024, 11, e2403388. 10.1002/advs.202403388 PMC11425287 39033533

[19] S. Dong , Y. Dong , T. Jia , S. Liu , J. Liu , D. Yang , F. He , S. Gai , P. Yang , J. Lin , Adv. Mater. 2020, 32, 2002439. 10.1002/adma.202002439 32914495

[20] T. S. Carpenter , S. M. Rosolina , Z. Xue , Sens. Actuators, B 2017, 253, 846.

[21] P. Binarová , J. Tuszynski , Cells 2019, 8, 1294. 31652491 10.3390/cells8101294 PMC6829893

[22] a) B. Jung , V. I. Vullev , B. Anvari , IEEE J. Sel. Top. Quantum Electron. 2013, 20, 149;

[23] E. K. H. Shea , V. C. Y. Koh , P. H. Tan , Pathol. Int. 2020, 70, 242. 32039524 10.1111/pin.12910

[24] E. L. Vos , J. Gaal , C. Verhoef , K. Brouwer , C. H. M. van Deurzen , L. B. Koppert , Eur. J. Surg. Oncol. 2017, 43, 1846. 28688723 10.1016/j.ejso.2017.06.007

[25] D. Gao , Z. Luo , Y. He , L. Yang , D. Hu , Y. Liang , H. Zheng , X. Liu , Z. Sheng , Small 2023, 19, 2206544. 10.1002/smll.202206544 36710248

[26] R. Yang , P. Wang , K. Lou , Y. Dang , H. Tian , Y. Li , Y. Gao , W. Huang , Y. Zhang , X. Liu , G. Zhang , Adv. Sci. 2022, 9, 2104728. 10.1002/advs.202104728 PMC9036023 35170876

[27] M. Wei , J. Bai , X. Shen , K. Lou , Y. Gao , R. Lv , P. Wang , X. Liu , G. Zhang , ACS Nano 2023, 17, 11345. 37272787 10.1021/acsnano.3c00350 PMC10311599

[28] S. Bonvalot , P. L. Rutkowski , J. Thariat , S. Carrère , A. Ducassou , M.‐P. Sunyach , P. Agoston , A. Hong , A. Mervoyer , M. Rastrelli , V. Moreno , R. K. Li , B. Tiangco , A. C. Herraez , A. Gronchi , L. Mangel , T. Sy‐Ortin , P. Hohenberger , T. de Baère , A. Le Cesne , S. Helfre , E. Saada‐Bouzid , A. Borkowska , R. Anghel , A. Co , M. Gebhart , G. Kantor , A. Montero , H. H. Loong , R. Vergés , et al., Lancet Oncol. 2019, 20, 1148. 31296491 10.1016/S1470-2045(19)30326-2

