# Supplementary material for: A Hf(IV)‐Coordinated NIR‐II Fluorescence “Turn‐On” Nanoprobe for Precise Imaging‐Guided Surgery in Breast Cancer
Source: Adv Sci (Weinh). 2025 Feb 14;12(14):2413385. doi: 10.1002/advs.202413385 (PMC11984921; doi:10.1002/advs.202413385)
Supplement: Supplementary file 1 — Supporting Information [file ADVS-12-2413385-s001.docx]

Supporting Information

**A Hf(IV)-coordinated NIR-II fluorescence “turn-on” nanoprobe for precise imaging-guided surgery in breast cancer**

*Yueyang He^#^, Jinyan Lin^#^, Jingwen Bai^#^, Xiao Shen, Kangliang Lou, Yuanyuan Zhu, Zishan Qiao, Weiling Chen, Yang Li^*^, Xiaolong Liu^*^, Guojun Zhang^*^*


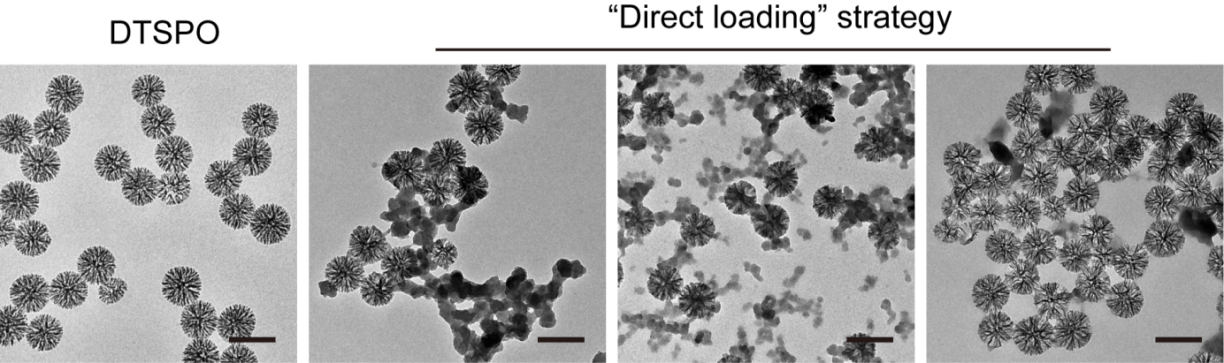


**Figure S1.** TEM images of DTSPO and immature IHD. Immature IHD nanoparticles were synthesized by “direct loading” strategy for comparison with IHD nanoprobes synthesized by “controllable space-limited coordination” strategy. TEM images were acquired from three parallel experiments. Scale bars = 200 nm.


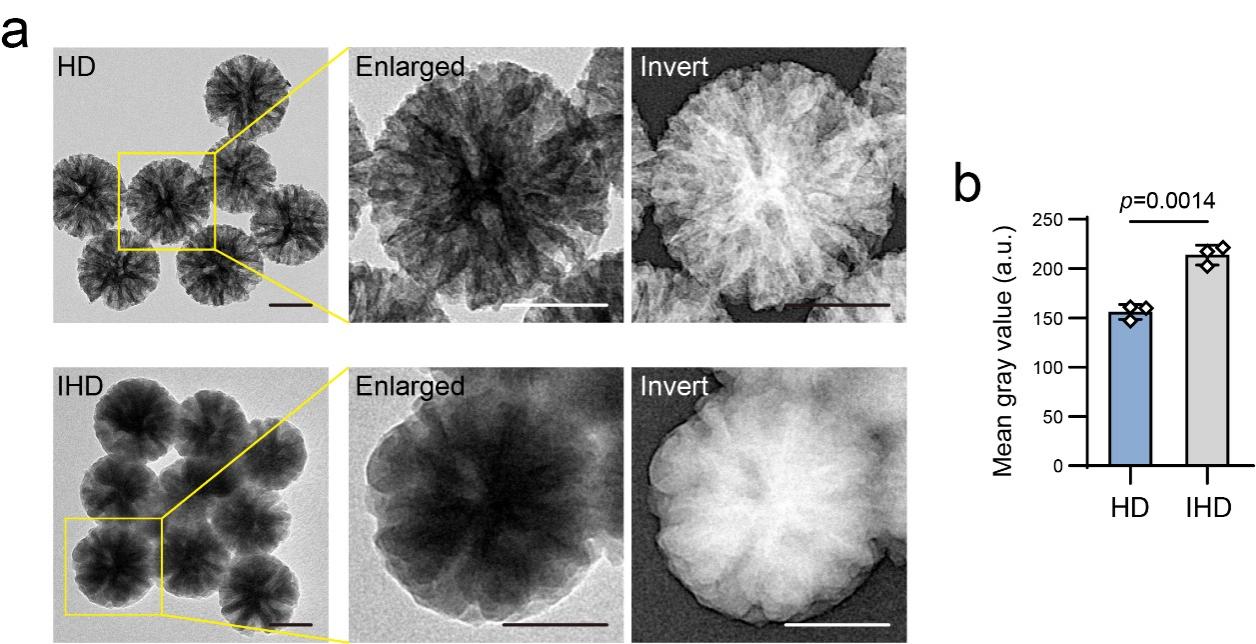


**Figure S2.** a) Enlarged TEM images of HD and IHD originated from Figure 1a. b) Corresponding quantitative analysis of Figure S2a. Scale bars = 100 nm.


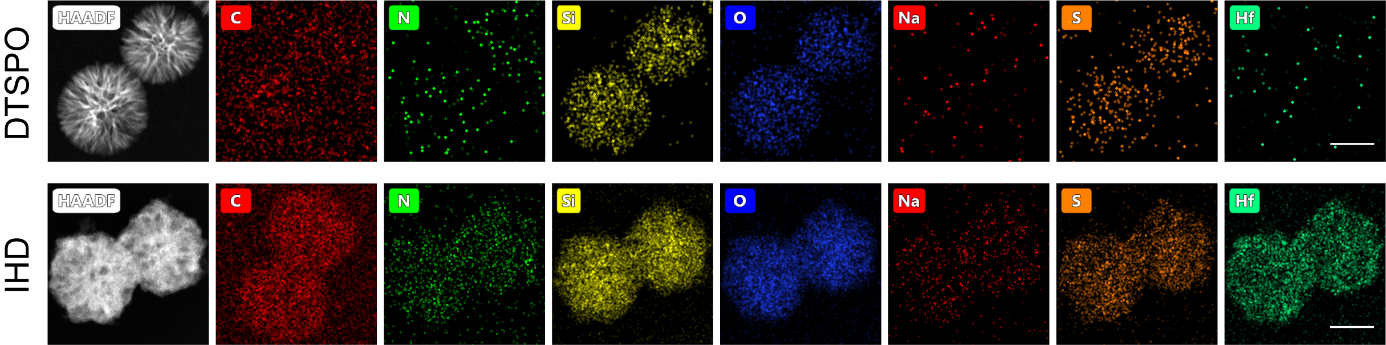


**Figure S3.** HAADF images along with element mapping images (C, N, Si, O, Na, S, and Hf) of DTSPO and IHD. Scale bars = 100 nm.

.
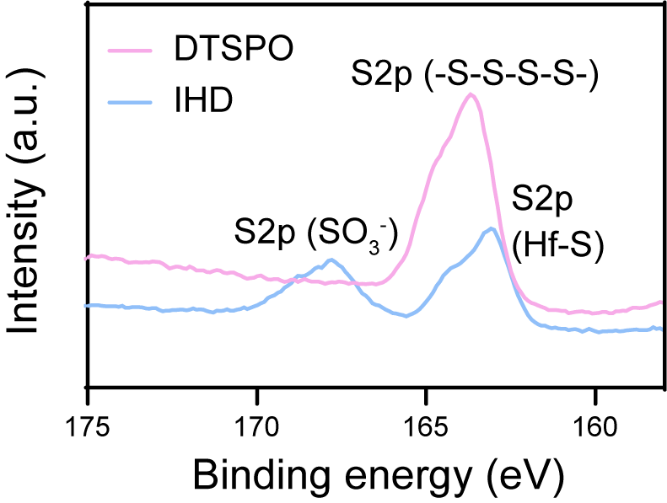


**Figure S4.** XPS spectrum in S2p region of IHD.


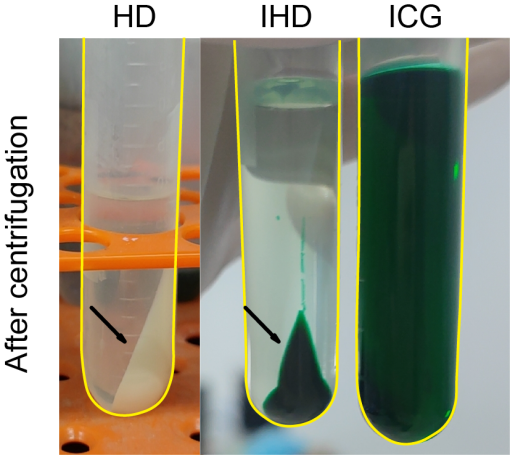


**Figure S5.** Photograph of HD, IHD, and ICG after centrifugation.


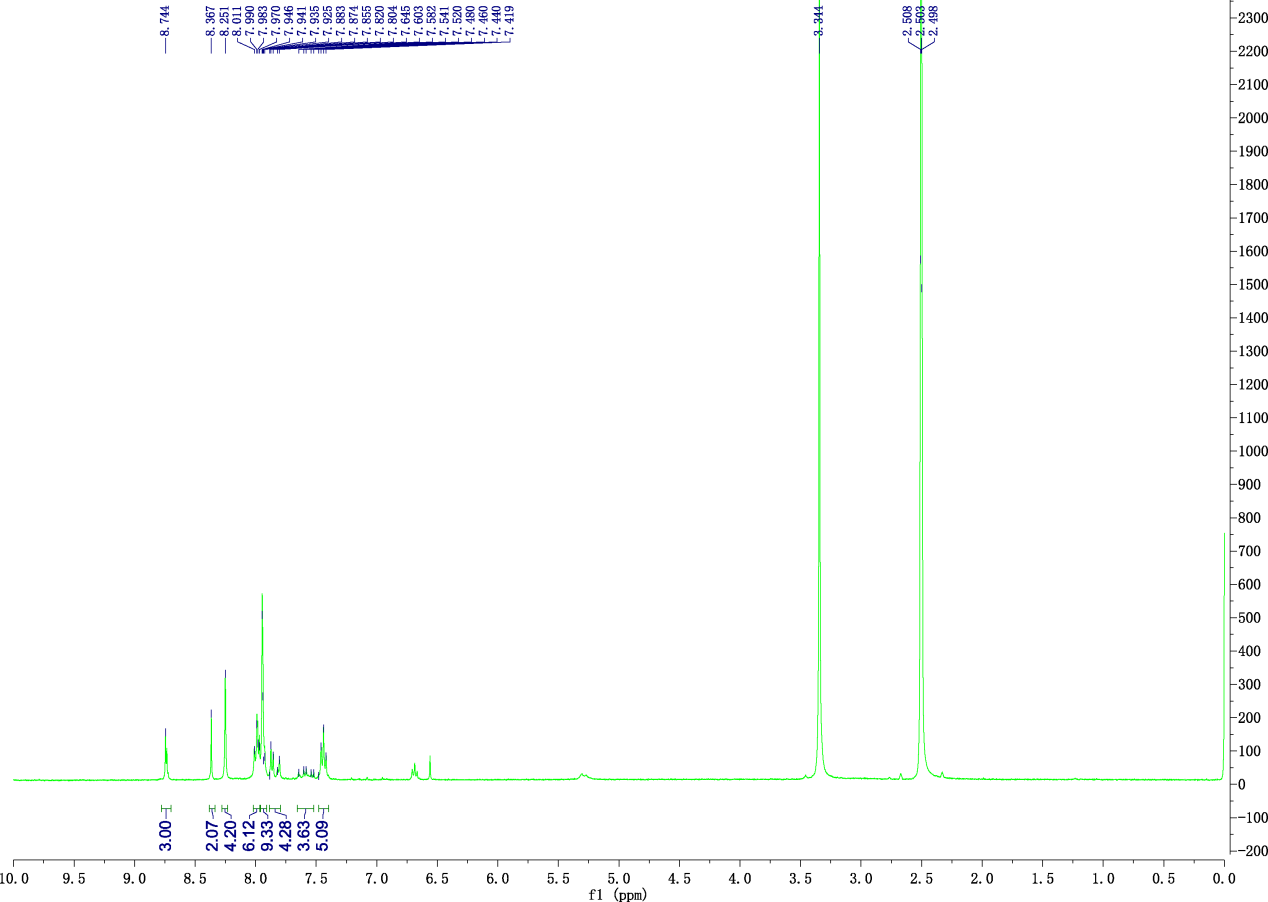


**Figure S6.** ^1^H NMR spectrum of TAB. ^1^H NMR (400 MHz, DMSO-*d*_6_) δ (ppm): 8.74 (s, 3H), 8.36 (s, 2H), 8.25 (s, 4H), 8.01-7.97 (m, 6H), 7.95-7.92 (m, 9H), 7.88-7.80 (m, 4H), 7.64-7.52 (m, 3H), 7.48-7.42 (d, 5H).


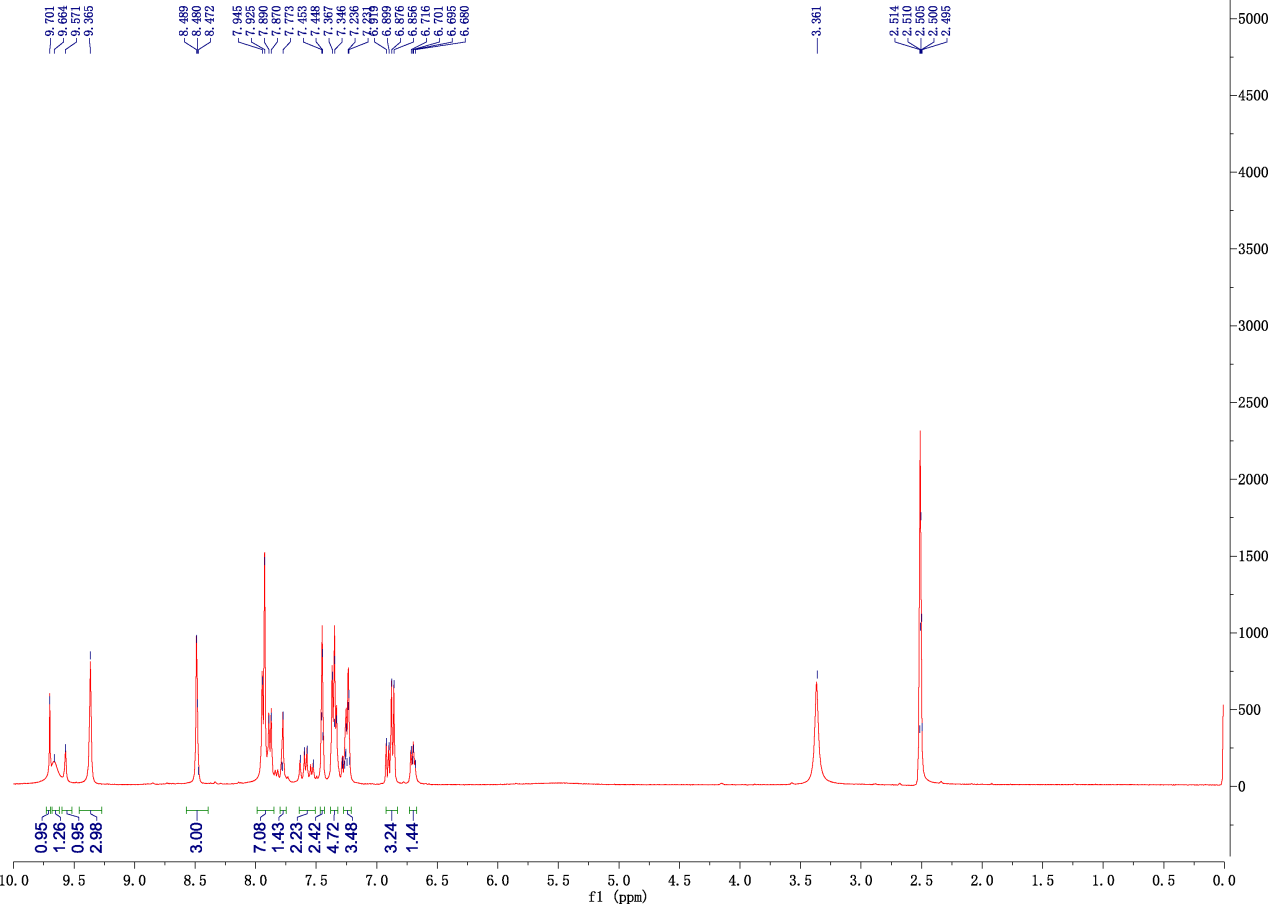


**Figure S7.** ^1^H NMR spectrum of TAC. ^1^H NMR (400 MHz, DMSO-*d*_6_) δ (ppm): 9.70 (s, 1H), 9.66 (s, 1H), 9.57 (s, 1H), 9.36 (s, 3H), 8.48 (s, 3H), 7.94-7.77 (m, 9H), 7.62-7.52 (m, 2H), 7.45 (s, 3H), 7.36-7.33 (m, 4H), 7.28-7.22 (m, 3H), 6.91-6.85 (m, 3H), 6.71-6.68 (m, 1H).


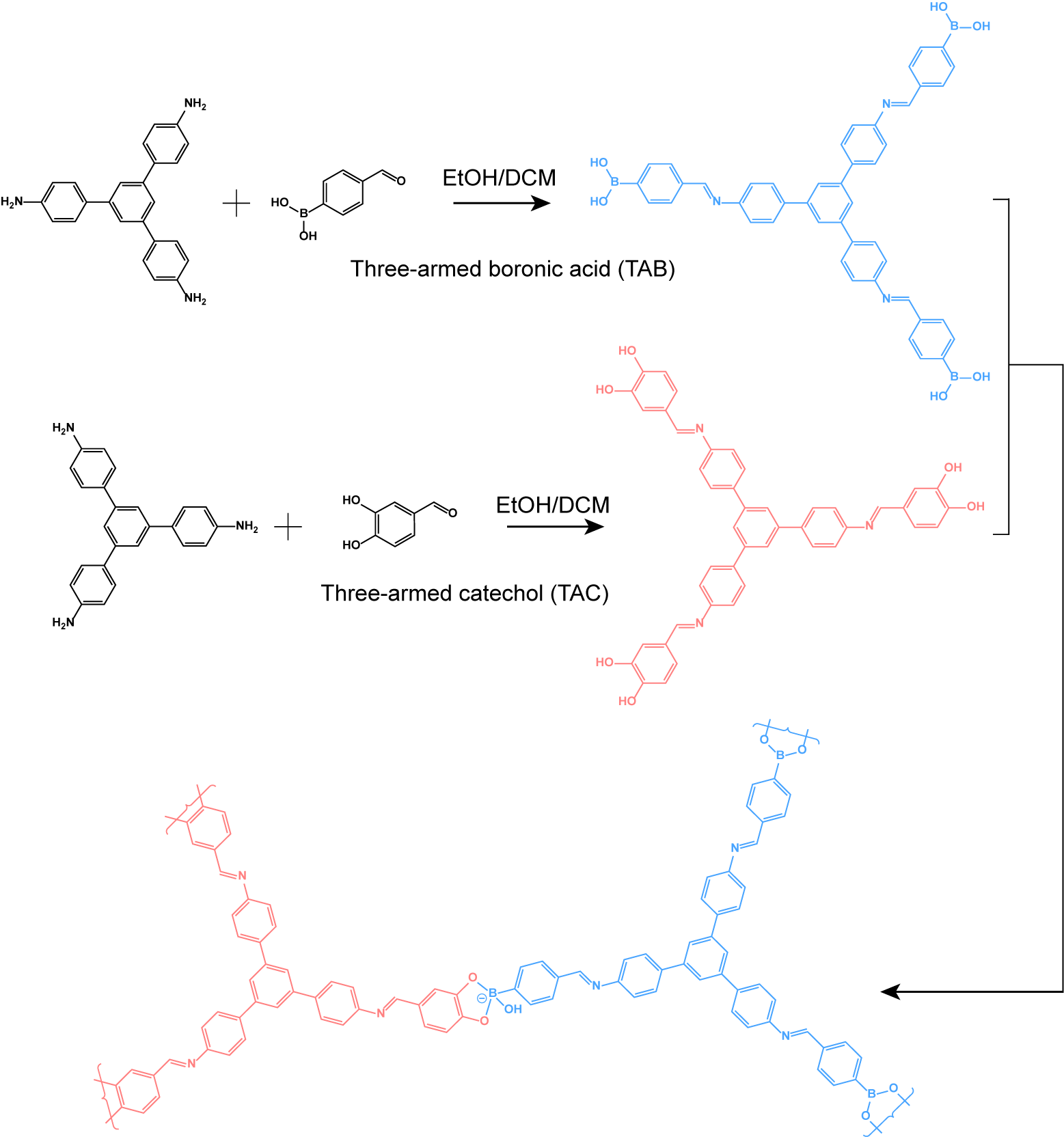


**Figure S8.** Synthesis routes of TAB, TAC, and TAC-TAB crosslinking.


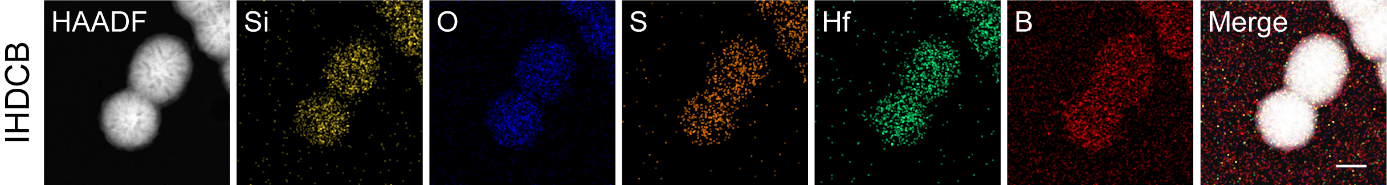


**Figure S9.** HAADF images along with element mapping images (Si, O, S, Hf, and B) of IHDCB. Scale bars = 100 nm.


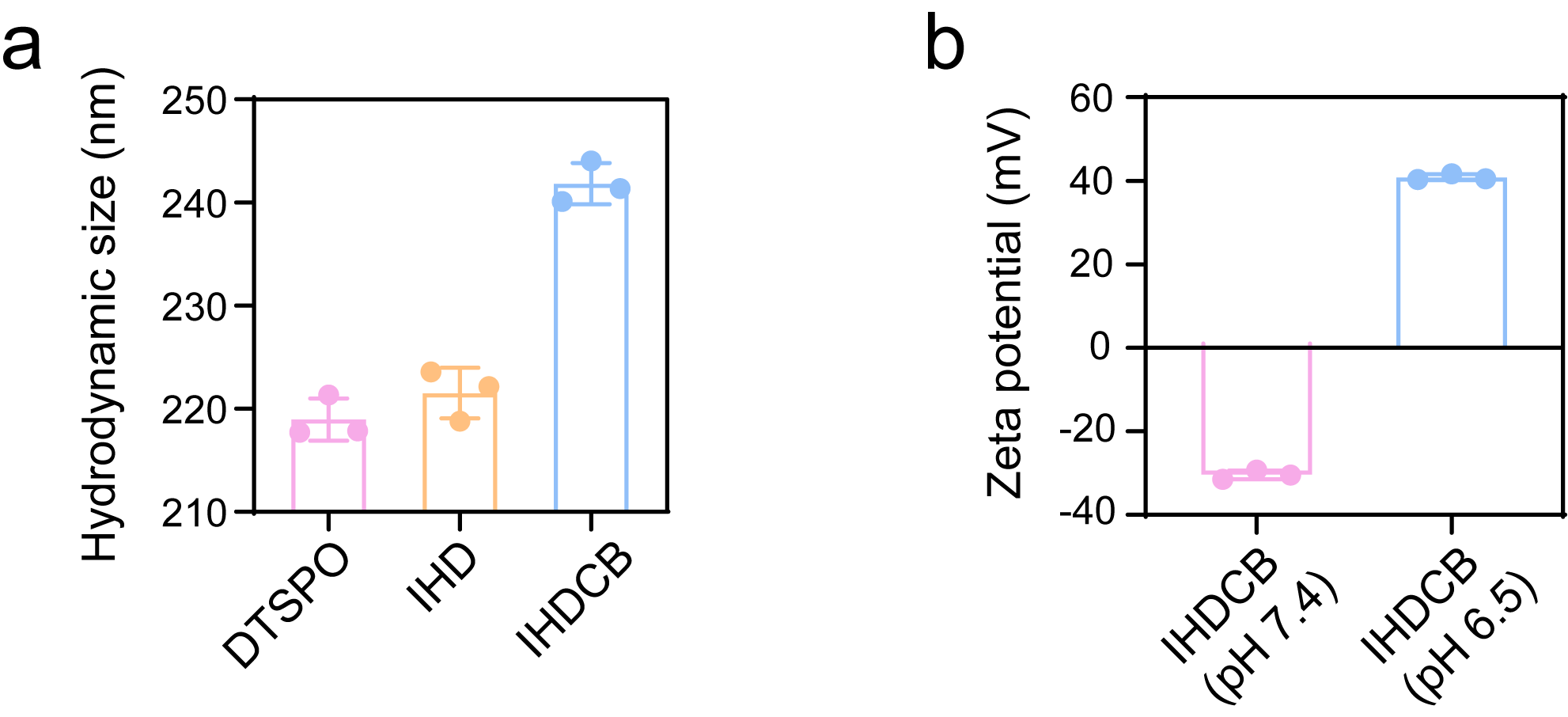


**Figure S10.** a) The hydrodynamic diameters of DTSPO, IHD, and IHDCB (*n* = 3). b) The zeta potential of IHDCB at pH 7.4 and pH 6.5 (*n* = 3). Data are presented as mean values ± SD.


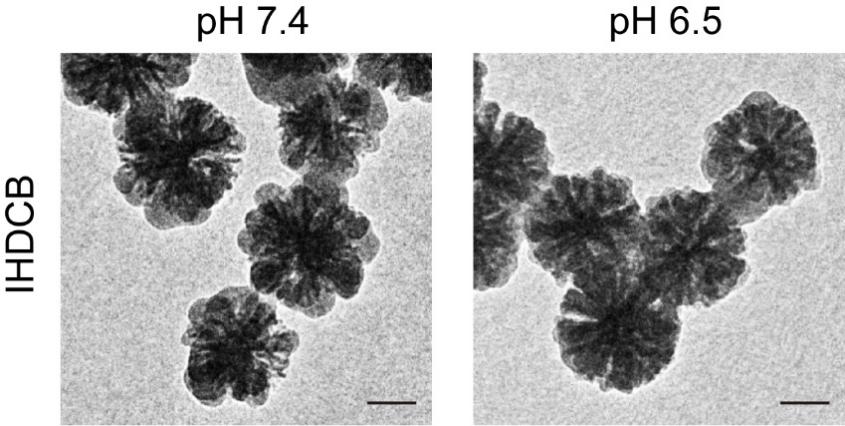


**Figure S11.** TEM images of IHDCB at pH 7.4 and pH 6.5. Scale bars = 100 nm.


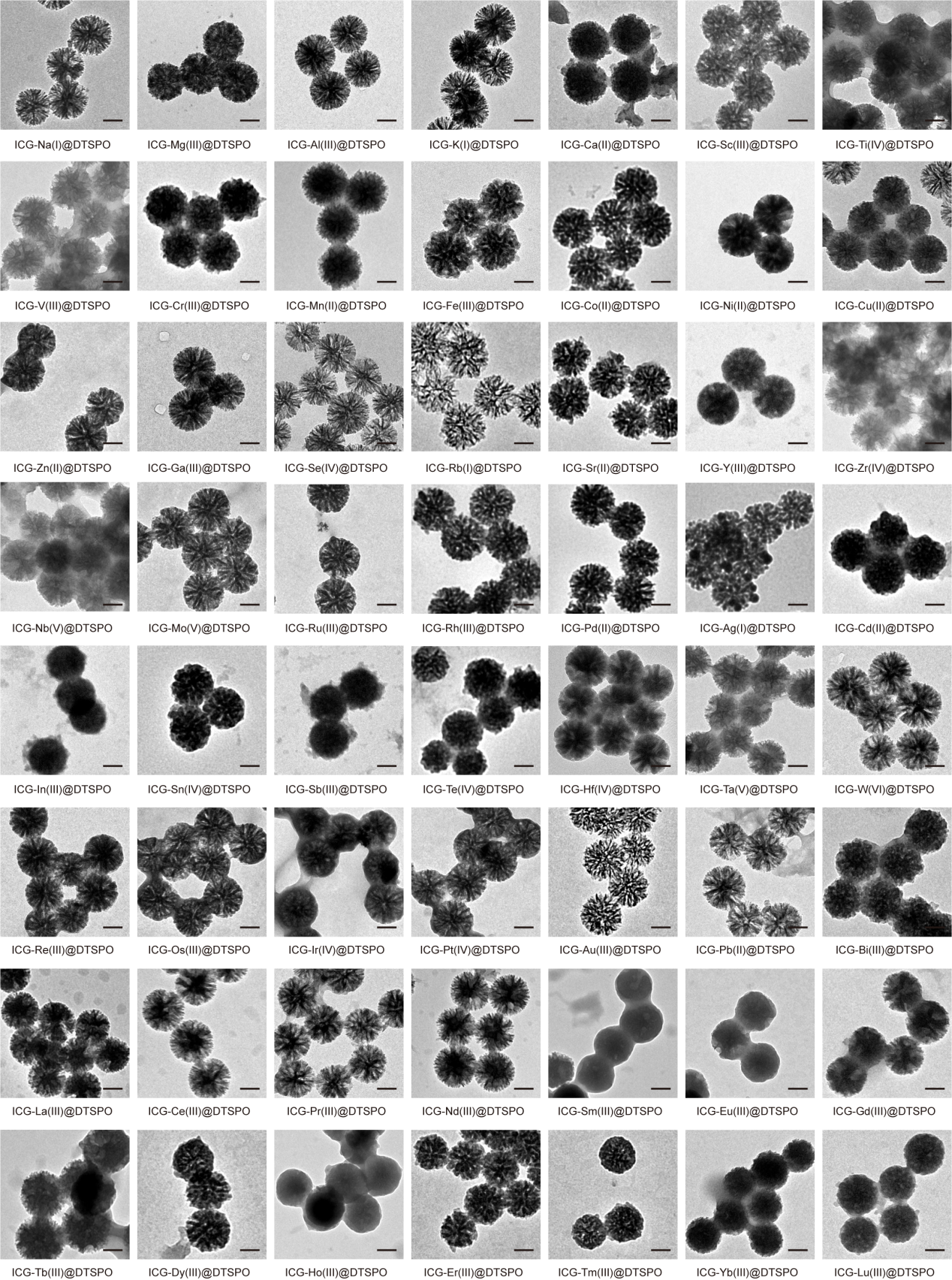


**Figure S12.** TEM images of ICG-X@DTSPO (X: Na, Mg, Al, K, Ca, Sc, Ti, V, Cr, Mn, Fe, Co, Ni, Cu, Zn, Ga, Se, Rb, Sr, Y, Zr, Nb, Mo, Ru, Rh, Pd, Ag, Cd, In, Sn, Sb, Te, Hf, Ta, W, Re, Os, Ir, Pt, Au, Pb, Bi, La, Ce, Pr, Nd, Sm, Eu, Gd, Tb, Dy, Ho, Er, Tm, Yb, and Lu). Scale bars = 100 nm. TEM image of ICG-Hf(IV)@DTSPO was originated from Figure 1a, in which ICG-Hf(IV)@DTSPO was denoted as abbreviation IHD.


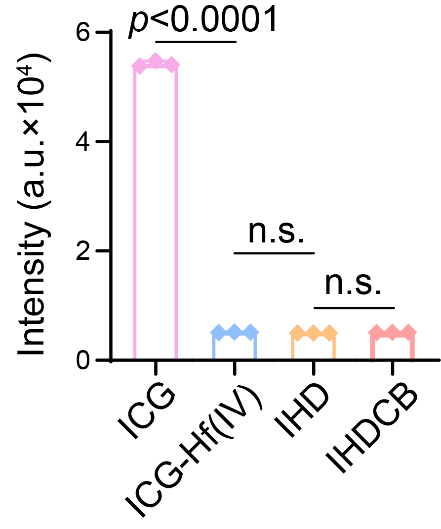


**Figure S13.** Fluorescence intensity of ICG, ICG-Hf(IV), IHD, and IHDCB at the same ICG concentration (*n* = 3). Data are presented as mean values ± SD. Statistical significance was calculated via one-way ANOVA with Tukey’s multiple comparisons test.


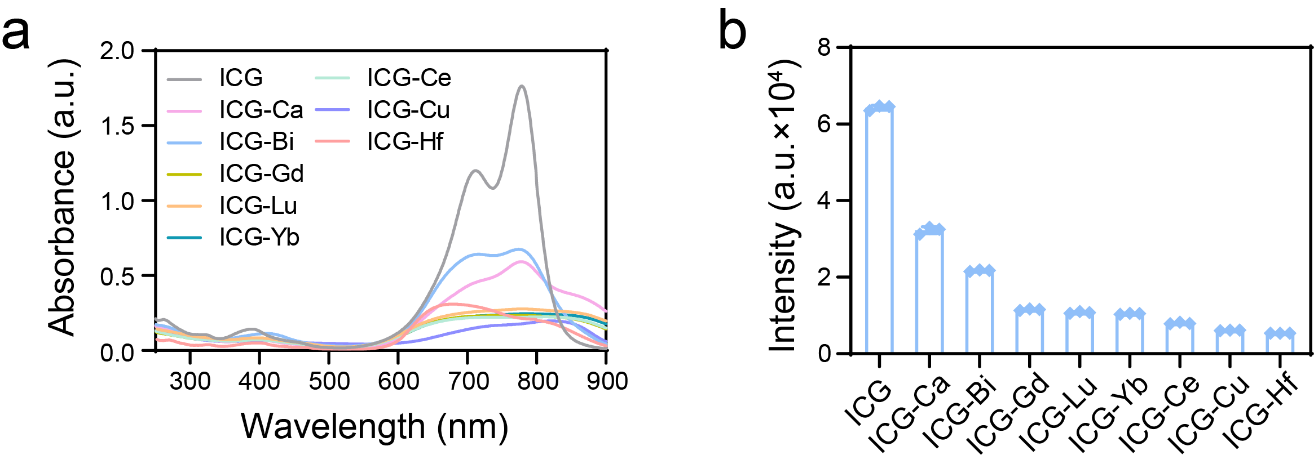


**Figure S14.** a) UV-vis-NIR absorption spectra of ICG and ICG-X (X: Ca, Bi, Gd, Lu, Yb, Ce, Cu, and Hf) coordination compounds. b) Fluorescence intensity of ICG and ICG-X (X: Ca, Bi, Gd, Lu, Yb, Ce, Cu, and Hf) coordination compounds (*n* = 3). Data are presented as mean values ± SD.


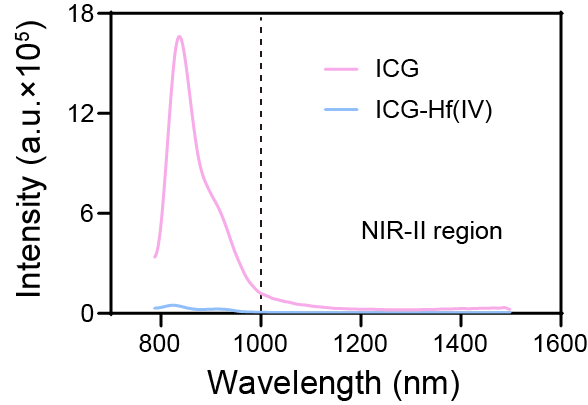


**Figure S15.** Enlarged fluorescence spectra of ICG and ICG-Hf(IV) in Figure 2d.


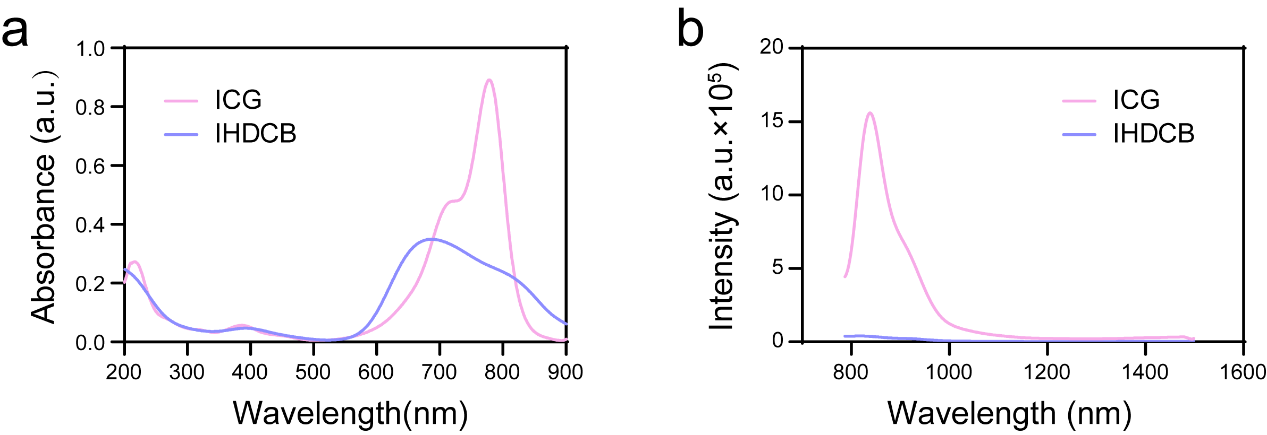


**Figure S16.** a) UV-vis-NIR absorption spectra of ICG and IHDCB. b) Fluorescence spectra of ICG and IHDCB.


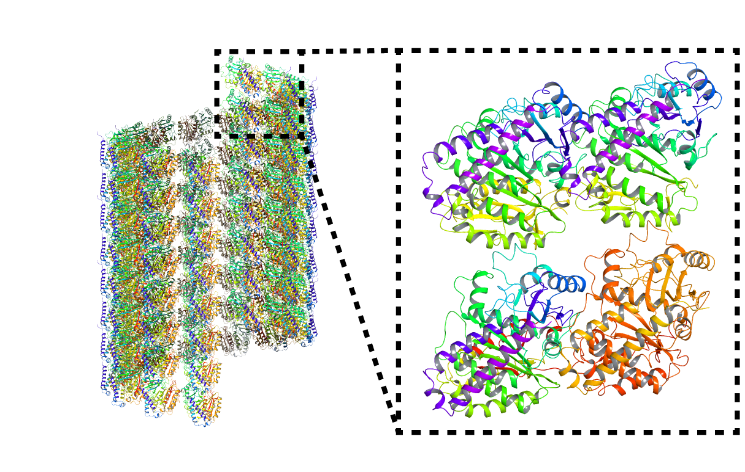


**Figure S17.** Structural schematic of tubulin.


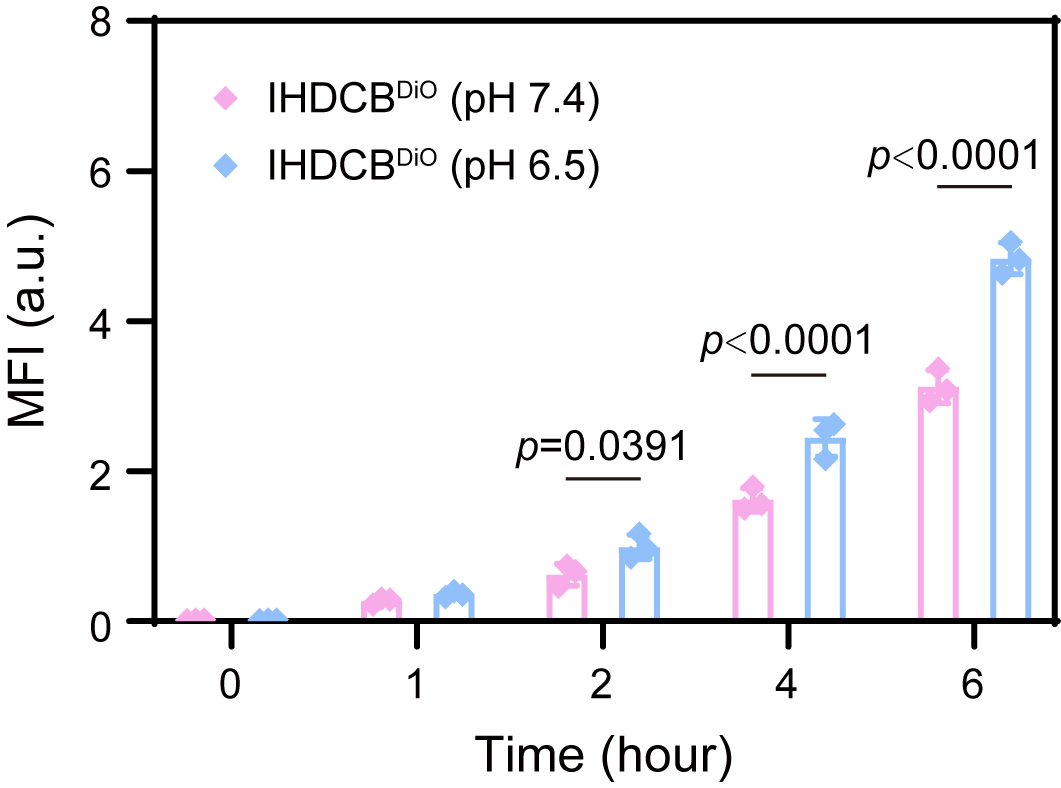


**Figure S18.** The MFI of DiO in different groups in Figure 3d (*n* = 3). Data are presented as mean values ± SD. Statistical significance was calculated via two-way ANOVA with Sidak's multiple comparisons test.


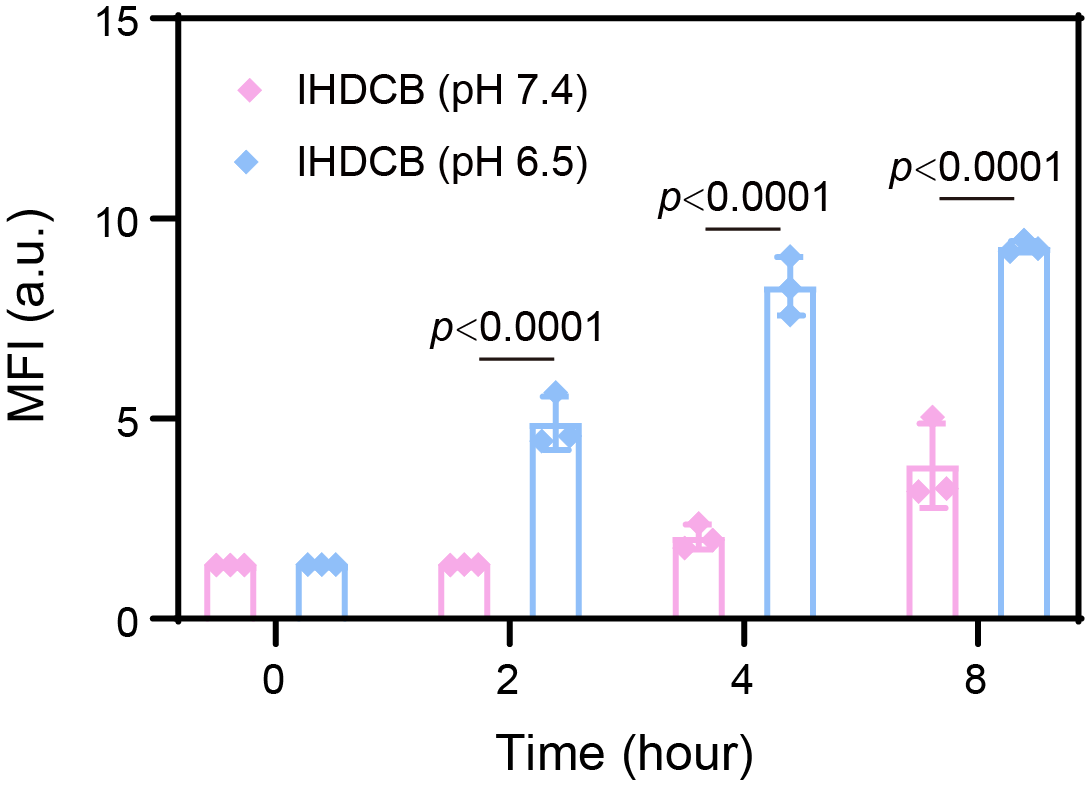


**Figure S19.** The MFI of ICG in different groups in Figure 3f (*n* = 3). Data are presented as mean values ± SD. Statistical significance was calculated via two-way ANOVA with Sidak's multiple comparisons test.


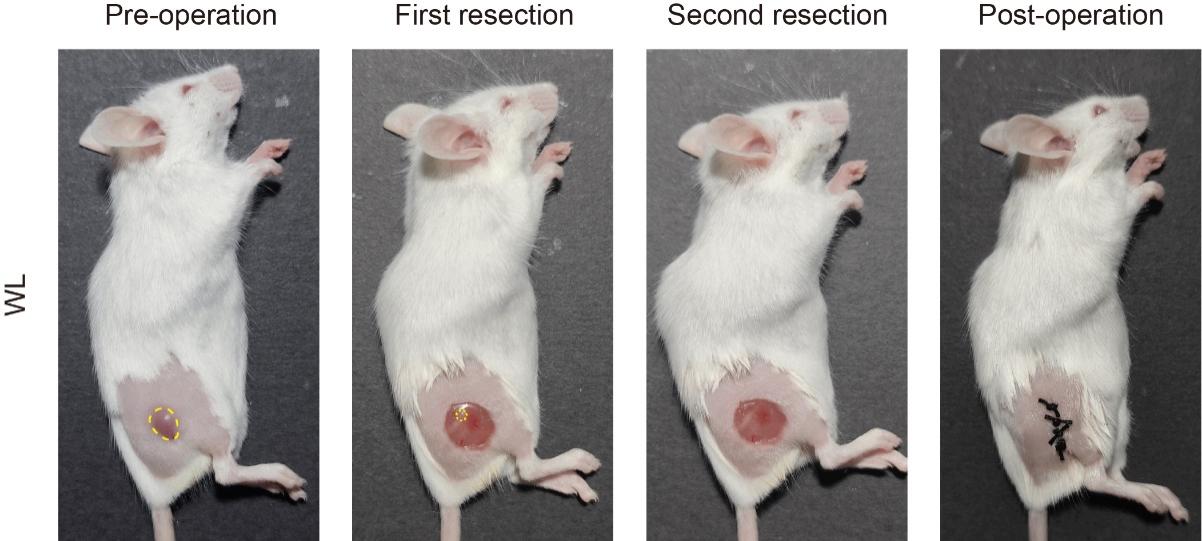


**Figure S20.** The representative preoperative, intraoperative (after first resection and second resection), and postoperative images under white light during the process of NIR-II fluorescence imaging-guided surgery in mice bearing subcutaneous 4T1-Luc tumor.

**
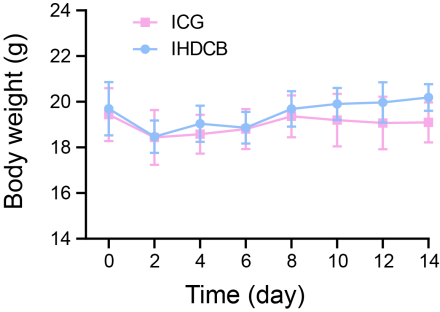
**

**Figure S21.** Body weight curve of mice through the course of NIR-II fluorescence imaging-guided subcutaneous tumor resection surgery (*n* = 6 mice). Data are presented as mean values ± SD.

**
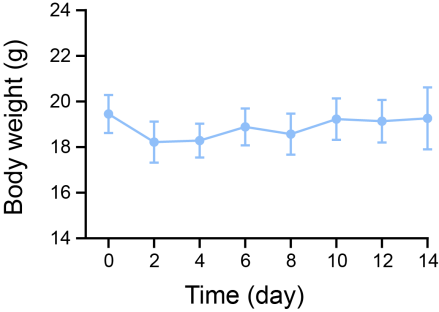
**

**Figure S22.** Body weight curve of mice through the course of NIR-II fluorescence imaging-guided muscle-infiltrating tumor resection surgery (*n* = 6 mice). Data are presented as mean values ± SD.


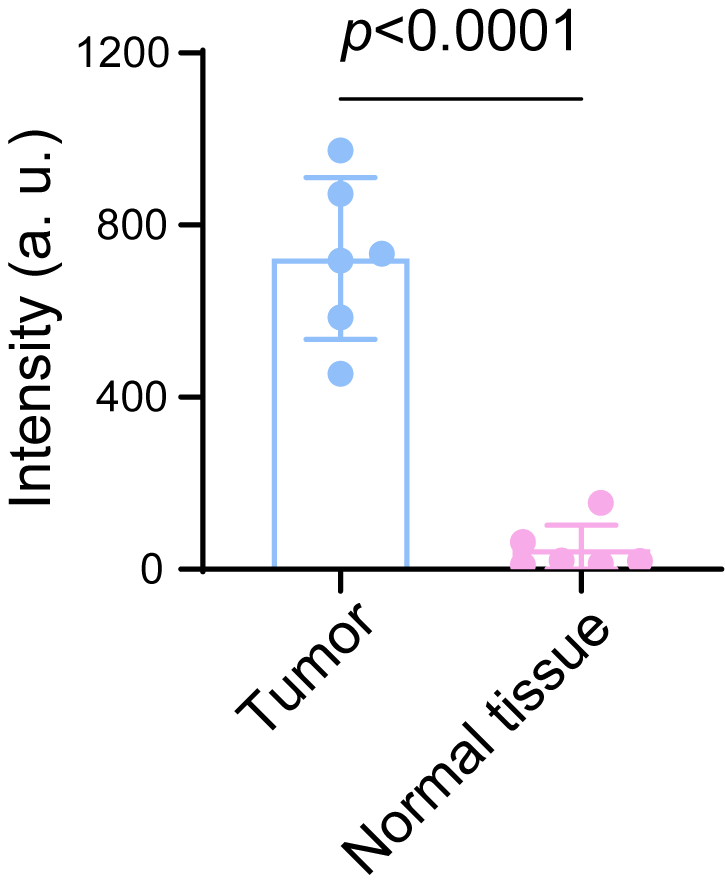


**Figure S23.** The fluorescence intensity of tumor and normal tissue sites consistent with H&E results in NIR-II images of Figure 7f (*n* = 6). Data are presented as mean values ± SD. Statistical significance was calculated via two-tailed unpaired t-test.


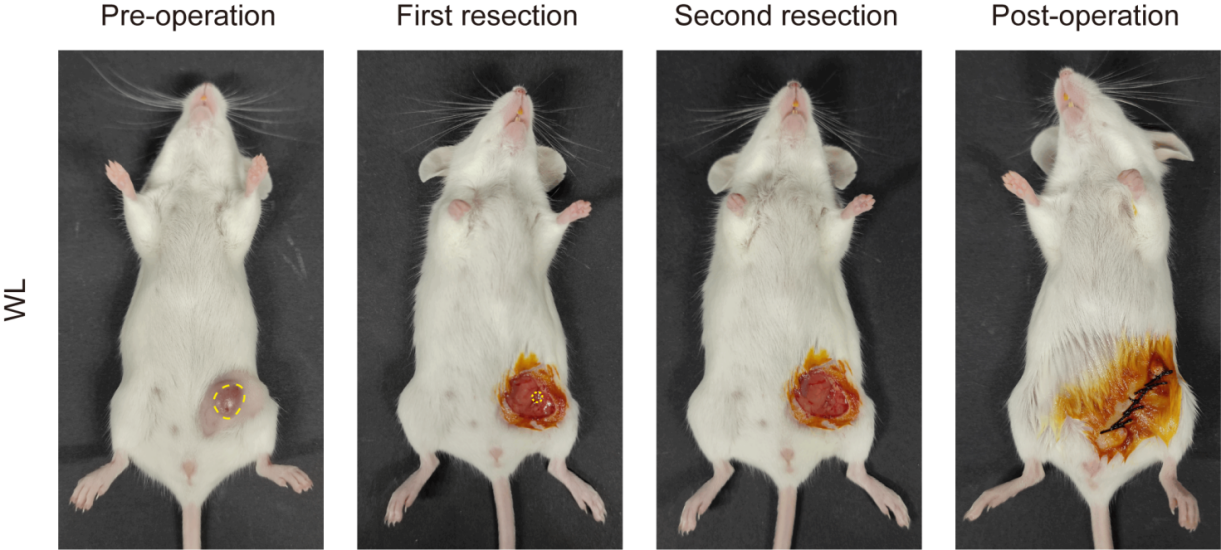


**Figure S24.** The representative preoperative, intraoperative (after first resection and second resection), and postoperative images under white light during the process of NIR-II fluorescence imaging-guided surgery in mice bearing orthotopic 4T1-Luc tumor.


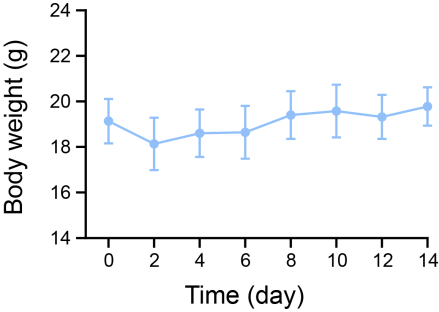


**Figure S25.** Body weight curve of mice through the course of NIR-II fluorescence imaging-guided orthotopic tumor resection surgery (*n* = 6 mice). Data are presented as mean values ± SD.


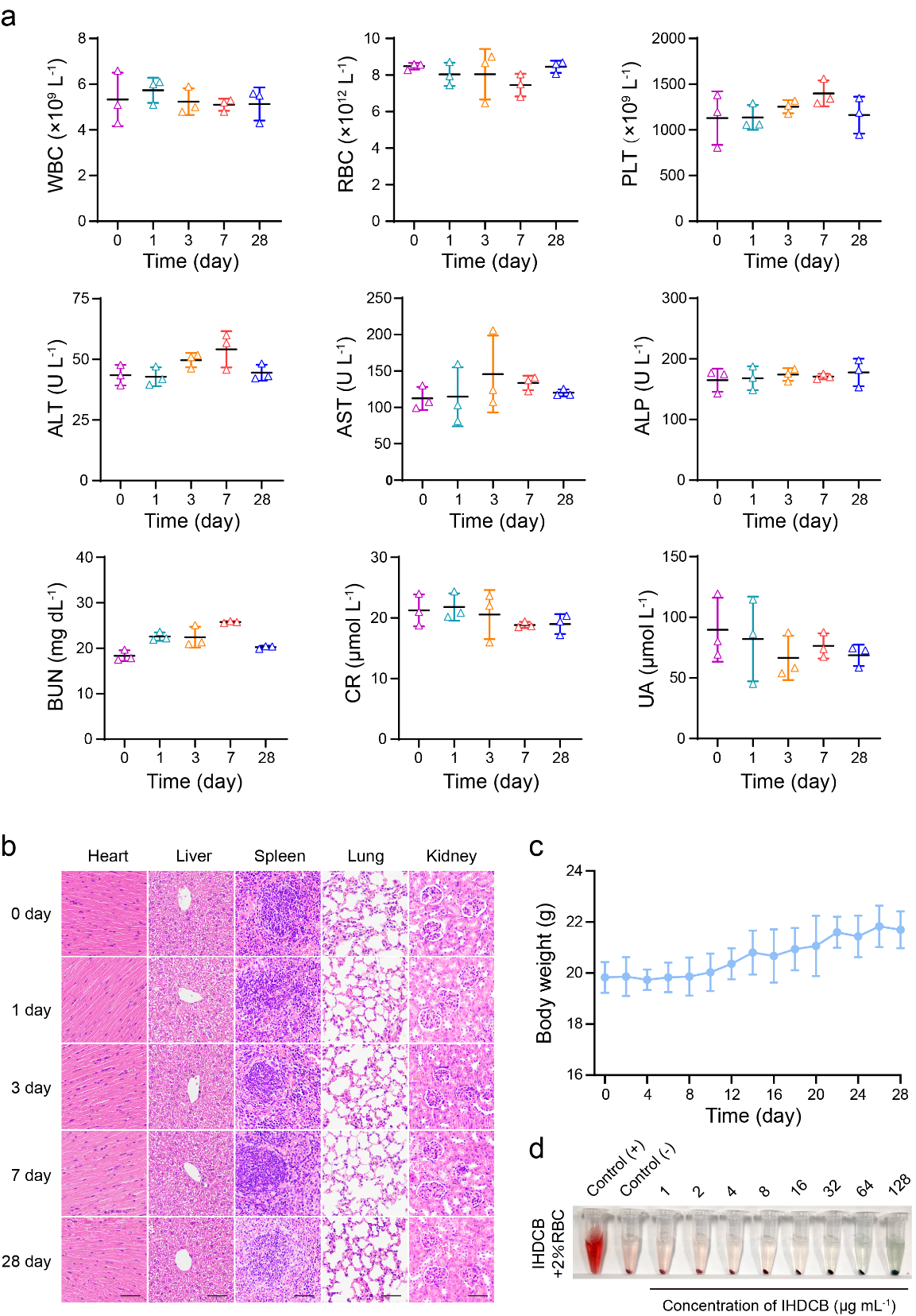


**Figure S26.** Biosafety evaluation. a) Assessment of the blood routine (WBC, RBC, and PLT) (*n* = 3 mice) and blood biochemical variables (ALT, AST, ALP, BUN, CR, and UA) (*n* = 3 mice) after intravenous injection of IHDCB for 0, 1, 3, 7, and 28 days. b) Representative H&E staining images of the main organs of BALB/c mice after intravenous injection of IHDCB for 0, 1, 3, 7, and 28 days (*n* = 3 mice). Scale bars = 50 μm. c) Body weight curve of mice after intravenous injection of IHDCB (*n* = 3 mice). d) Hemolysis assay of various concentrations of IHDCB after co-incubation with mouse red blood cells, and then imaged by digital camera. Data are presented as mean values ± SD.


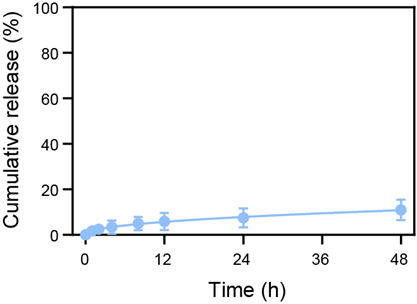


**Figure S27.** Cumulative release profiles of DiO for DiO-labeled IHDCB (*n* = 3). Data are presented as mean values ± SD.
